# Configuration of active site segments in lytic polysaccharide monooxygenases steers oxidative xyloglucan degradation

**DOI:** 10.1186/s13068-020-01731-x

**Published:** 2020-05-29

**Authors:** Peicheng Sun, Christophe V. F. P. Laurent, Stefan Scheiblbrandner, Matthias Frommhagen, Dimitrios Kouzounis, Mark G. Sanders, Willem J. H. van Berkel, Roland Ludwig, Mirjam A. Kabel

**Affiliations:** 1 Laboratory of Food Chemistry, Wageningen University & Research, Bornse Weilanden 9, 6708 WG Wageningen, The Netherlands; 2 Biocatalysis and Biosensing Laboratory, Department of Food Science and Technology, BOKU-University of Natural Resources and Life Sciences, Vienna, Muthgasse 18, 1190 Vienna, Austria; 3 Institute of Molecular Modelling and Simulation, Department of Material Sciences and Process Engineering, BOKU-University of Natural Resources and Life Sciences, Vienna, Muthgasse 18, 1190 Vienna, Austria

**Keywords:** Plant cell wall, Lignocellulose, Biomass, Biorefinery, Hemicellulose, Xyloglucan, AA9 LPMO, *Neurospora crassa*, Active site segments, Phylogenetic tree

## Abstract

**Background:**

Lytic polysaccharide monooxygenases (LPMOs) are powerful enzymes that oxidatively cleave plant cell wall polysaccharides. LPMOs classified as fungal Auxiliary Activities family 9 (AA9) have been mainly studied for their activity towards cellulose; however, various members of this AA9 family have been also shown to oxidatively cleave hemicelluloses, in particularly xyloglucan (XG). So far, it has not been studied in detail how various AA9 LPMOs act in XG degradation, and in particular, how the mode-of-action relates to the structural configuration of these LPMOs.

**Results:**

Two *Neurospora crassa* (*Nc*) LPMOs were found to represent different mode-of-action towards XG. Interestingly, the configuration of active site segments of these LPMOs differed as well, with a shorter Segment 1 (^−^Seg1) and a longer Segment 2 (^+^Seg2) present in *Nc*LPMO9C and the opposite for *Nc*LPMO9M (^+^Seg1^−^Seg2). We confirmed that *Nc*LPMO9C cleaved the non-reducing end of unbranched glucosyl residues within XG via the oxidation of the C4-carbon. In contrast, we found that the oxidative cleavage of the XG backbone by *Nc*LPMO9M occurred next to both unbranched and substituted glucosyl residues. The latter are decorated with xylosyl, xylosyl–galactosyl and xylosyl–galactosyl–fucosyl units. The relationship between active site segments and the mode-of-action of these *Nc*LPMOs was rationalized by a structure-based phylogenetic analysis of fungal AA9 LPMOs. LPMOs with a ^−^Seg1^+^Seg2 configuration clustered together and appear to have a similar XG substitution-intolerant cleavage pattern. LPMOs with the ^+^Seg1^−^Seg2 configuration also clustered together and are reported to display a XG substitution-tolerant cleavage pattern. A third cluster contained LPMOs with a ^−^Seg1^−^Seg2 configuration and no oxidative XG activity.

**Conclusions:**

The detailed characterization of XG degradation products released by LPMOs reveal a correlation between the configuration of active site segments and mode-of-action of LPMOs. In particular, oxidative XG-active LPMOs, which are tolerant and intolerant to XG substitutions are structurally and phylogenetically distinguished from XG-inactive LPMOs. This study contributes to a better understanding of the structure–function relationship of AA9 LPMOs.

## Background

For establishing sustainable processes and a circular economy, plant biomass is an essential source for the production of fuels and chemicals, in particular, to replace fossil-based resources [1]. Plant biomass dry matter is mainly composed of plant cell wall polymers, which are present in the middle lamella, primary and secondary cell wall layers [2]. The primary cell wall is mainly built of pectin, cellulose and hemicellulosic xyloglucan (XG), while the secondary cell wall is mostly composed of cellulose, hemicellulosic xylan or mannan and the aromatic polymer lignin [3–6]. While in general dicotyledonous plant biomass dry matter is majorly composed of primary cell wall components, other species such as grasses and wood plant biomass dry matter are majorly composed of secondary cell wall components [3–6]. An important step in biomass-based processes is the release of fermentable carbohydrates. In the last decade, monocopper-dependent lytic polysaccharide monooxygenases (LPMOs) have been shown to assist glycosyl hydrolases as green and effective tools for biomass polysaccharide degradation [7–10]. In this research, we aimed to understand how LPMOs oxidatively cleave XG, and hypothesized that the mode-of-action of LPMOs towards XG correlates with their active site configuration.

Cellulose is a linear macromolecule composed of β-(1 → 4) linked d-Glc*p* units [4]. Due to the absence of side chains, cellulose forms crystalline (ordered) microfibrils via van der Waals force and hydrogen bonds [2, 5]. Like cellulose, XG has a backbone of β-(1 → 4) linked d-Glc*p* residues, which is further substituted via the C6 position by α-(1 → 6) linked d-Xyl*p* residues [2, 11]. The xylosyl units can be further substituted with β-(1 → 2) linked d-Gal*p* and, rarely, α-(1 → 3) linked l-Ara*f* residues. In addition, an l-Fuc*p* unit via an α-(1 → 2) linkage to d-Gal*p* also occurs [11–14]. The type and amount of XG substituents highly vary and, for example, depends on the plant species or tissue [13]. To simplify the complicated trivial names of individual XG oligosaccharides, Fry and coworkers developed an unambiguous nomenclature using one-letter codes to represent the XG structure (Table 1) [15]. Most XG structures have been defined as block-wise “XXXG”- and “XXGG”-types [16]. For example, XG from tamarind seed (TXG) and black currant (BCXG) have been shown to consist of “XXXG” repeating units, with partially β-d-Gal*p* substituted blocks (XXLG, XLXG and XLLG) [15, 17]. Furthermore, additional substitutions with fucosyl units were defined for BCXG (XXFG and XLFG) [17]. Fucosylated XG has been found in many plant sources from campanulids (i.e., carrot), while XG from grass species is not composed of fucosylated residues [11]. Other modifications have been found as well, for instance acetylation on galactosyl residues [13, 18], but are not further discussed in this study.

**Table 1:**
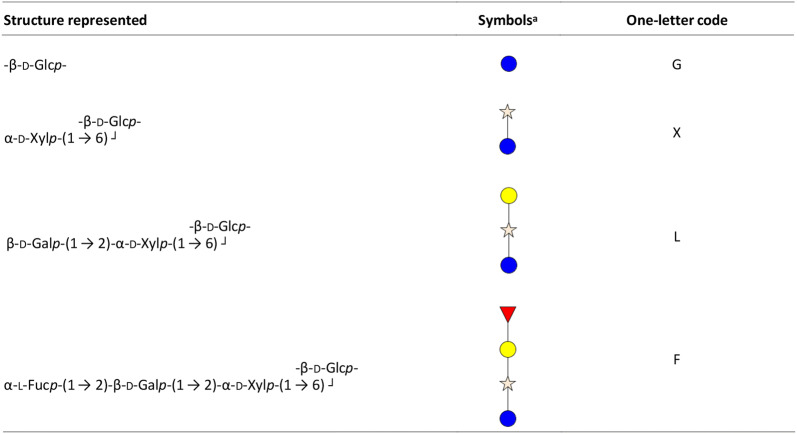
One-letter codes according to Fry et al. [15] and symbols used in this study for representing XG oligosaccharide structures

ablue circle: glucosyl unit; star: xylosyl unit; yellow circle: galactosyl unit; red triangle: fucosyl unit

LPMOs classified in the Carbohydrate-Active enzymes database (CAZy; http://www.cazy.org [19]) Auxiliary Activities family 9 (AA9) originate from fungi and have been shown to oxidatively cleave cellulose. Although less studied, for some AA9 members additional oxidative XG cleavage has been reported [20–32]. So far, oxidative XG cleavage of AA9 LPMOs has been mainly determined by the detection of formed oligosaccharides by using high-performance anion exchange chromatography with pulsed amperometric detection (HPAEC-PAD) and matrix-assisted laser desorption/ionization-time of flight mass spectrometry (MALDI-TOF-MS). Although these analytical techniques indicate an oxidative cleavage of XG, exact structures of released XG degradation products by AA9 LPMOs were not identified [20–33]. Nevertheless, from both studies it could be concluded that among the XG-active AA9 LPMOs generally two HPAEC-PAD patterns of TXG oligosaccharides have been shown: (i) generation of (block-wise) “XXXG”-type (oxidized) TXG oligosaccharides, e.g., *Mt*LPMO9J and *Nc*LPMO9C [22, 32]; (ii) generation of many different, non-“XXXG”-type (oxidized) TXG oligosaccharides, e.g., *Fg*LPMO9A and *Gt*LPMO9A-2 [27, 28]. To better understand these two suggested routes, a detailed product characterization is essential.

**Fig. 1:**
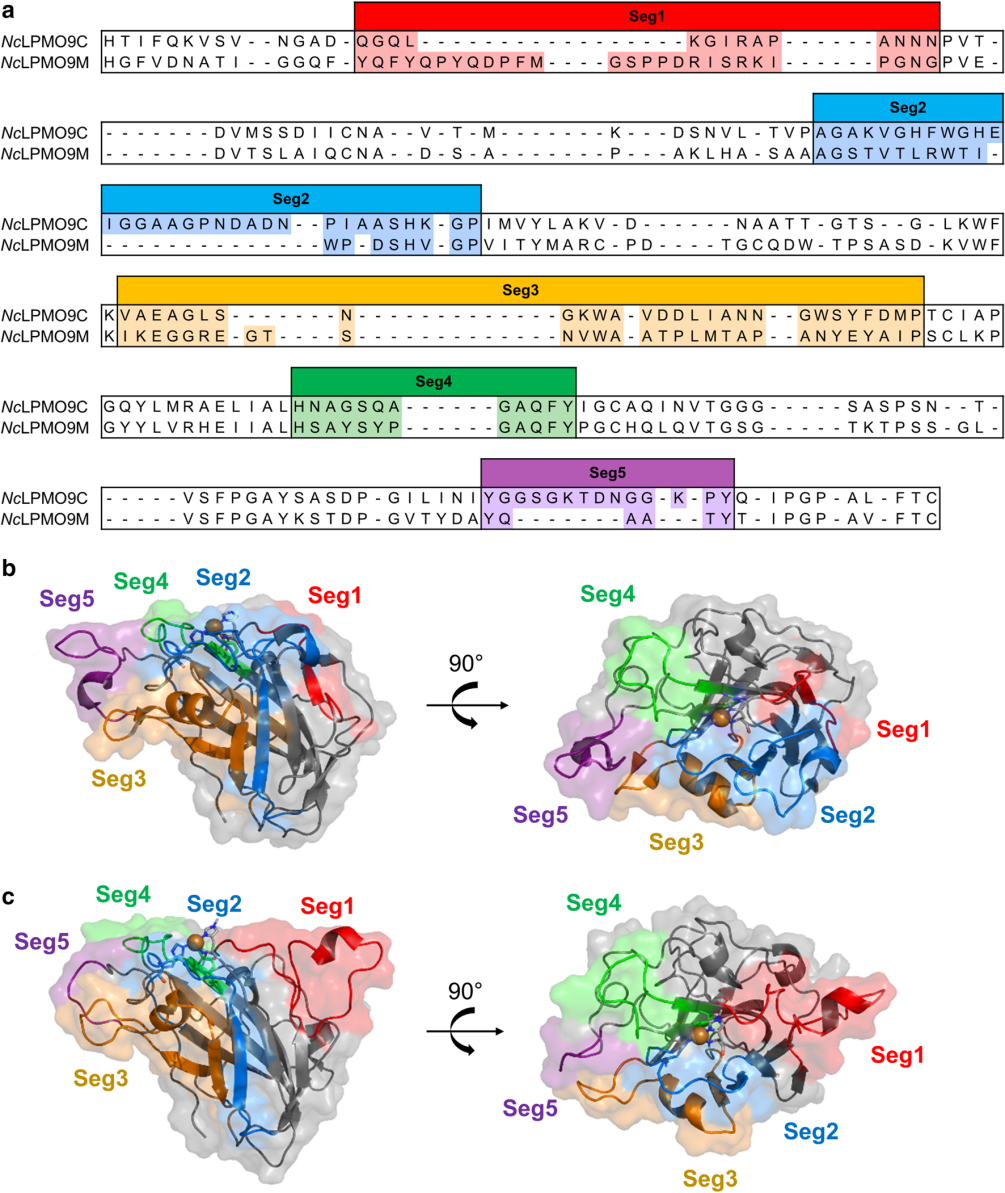
**a** Amino acid sequence alignments of *Nc*LPMO9C (PDB entry: 4D7U) and *Nc*LPMO9M (PDB entry: 4QI8) and **b**, **c** their respective crystal structures. The active site segments are indicated as Seg1 (red), Seg2 (blue), Seg3 (yellow), Seg4 (green) and Seg5 (purple)

Whether these different XG-cleavage pathways result from distinct XG-binding sites neighboring the catalytic site of the LPMOs has yet to be defined. Active site structures of LPMOs interacting with cellulosic substrates have already been reported [24, 34–38], but information about relevant binding sites of XG is still scarcely available in the literature. Courtade and coworkers have shown through NMR analysis that a so-called L3 loop around the active site of a XG-active *Nc*LPMO9C strongly interacted with XG [35]. This L3 loop also has been shown to be present in other XG-active LPMOs like *Pa*LPMO9H [31] and *Mt*LPMO9J [22]. However, in another XG-active *Gt*LPMO9A-2, the L3 loop is absent. Instead, *Gt*LPMO9A-2 has an extended L2 loop [28]. This difference might indicate that the configuration of segments around the AA9 LPMO active site influences their catalytic behavior on XG. The definition of the loops (L2, L3, LS and LC) around the active site has previously been suggested [38–40], and further redefined as segments in our previous study due to the presence of secondary structure elements [41]. Briefly, five segments (Seg1–Seg5) were defined, of which Seg1, Seg2, Seg3 and Seg5 are comparable, but slightly different, to the previously defined L2, L3, LS and LC regions, respectively (see also Fig. 1). Seg4 was newly defined and has not been described before.

In this work, two distinct product profiles of two different XG-active LPMOs from *Neurospora crassa* (*Nc*LPMO9C and *Nc*LPMO9M) were characterized by identification of the formed non-oxidized and oxidized XG oligosaccharides. In addition to various other chromatographic techniques, hydrophilic interaction chromatography coupled with electrospray ionization-collision induced dissociation-mass spectrometry (HILIC-ESI-CID-MS/MS) was used. To test our hypothesis that the mode-of-action of LPMOs towards XG is a result of their specific structural configuration around the active site, a structure-based sequence analysis of AA9 LPMOs was performed. The resulting phylogenetic tree shows three distinct groups, which not only differ in structural active site segments, but also seemingly correlate to the oxidative XG cleavage being either tolerant or intolerant to substitutions, and to XG-inactive LPMOs.

## Results

### *Nc*LPMO9C and *Nc*LPMO9M and their oxidative XG cleavage patterns

Two LPMOs from *N. crassa* with different active site segment configurations (*Nc*LPMO9C and *Nc*LPMO9M; Fig. 1) were tested for their mode-of-action towards XG. As presented in Fig. 1, *Nc*LPMO9C holds a short (^−^) Seg1 and a long (^+^) Seg2, whereas *Nc*LPMO9M has a ^+^Seg1^−^Seg2 configuration [41].

**Fig. 2:**
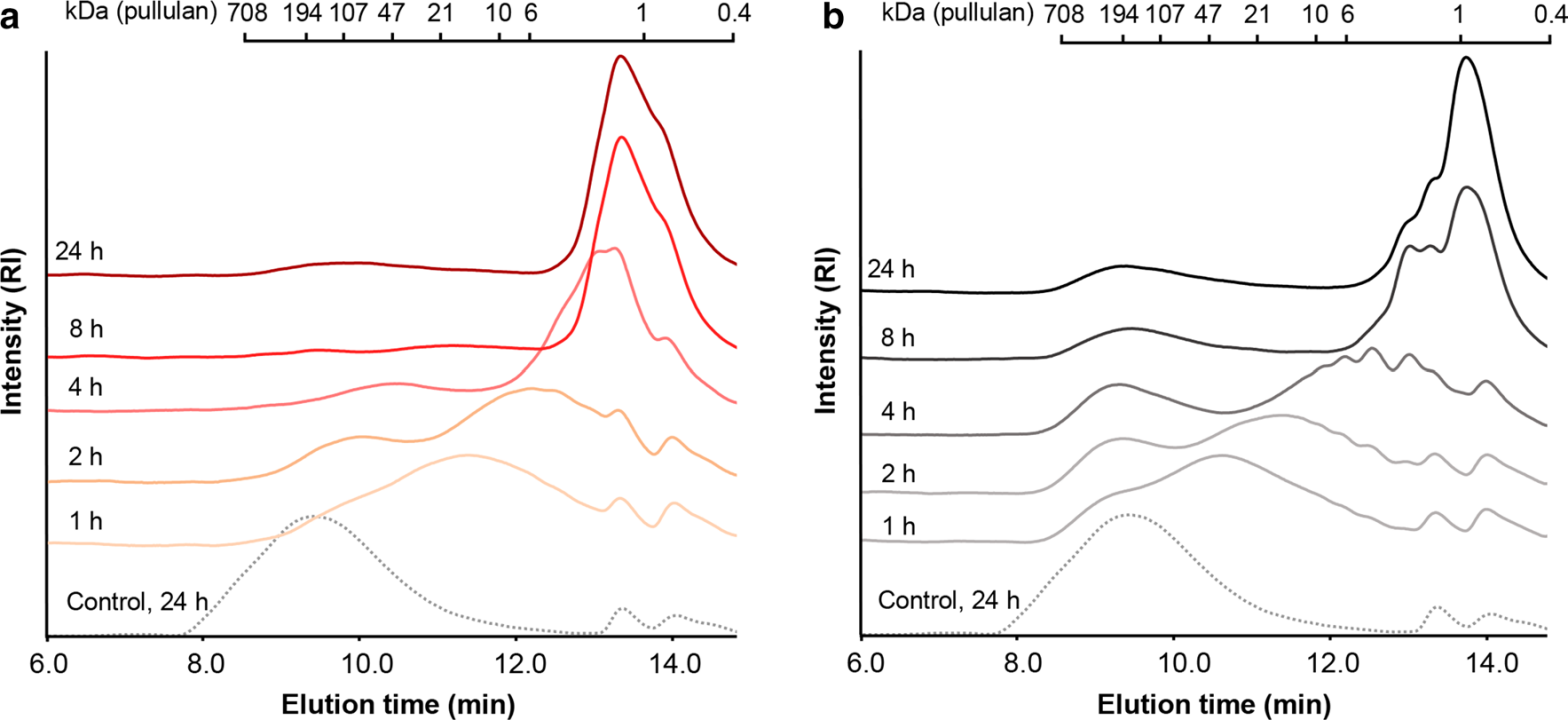
Molecular weight (MW) distribution of tamarind seed xyloglucan (TXG) digests in presence of ascorbic acid (Asc) from **a** 1.25 µM *Nc*LPMO9M and **b** 1.25 µM *Nc*LPMO9C, analyzed by HPSEC-RI. Dotted lines are control reactions containing only TXG with Asc. MW distributions of additional control digests are shown in Additional file 1: Fig. S1. MWs are indicated based on calibration with a series of pullulan standards (0.4–708 kDa)

We first monitored the mode-of-action of two *Nc*LPMOs on TXG by profiling the molecular weight (MW) distribution of *Nc*LPMO9M- and *Nc*LPMO9C-TXG-digests during incubation using high-performance size exclusion chromatography coupled to a refractive index detector (HPSEC-RI) (Fig. 2; Additional file 1: Fig. S1). The MW distribution of both *Nc*LPMO-TXG-digests after 24 h incubation showed only little change in the absence of ascorbic acid (Asc) (Additional file 1: Fig. S1), which showed that these enzyme preparations were almost free of hydrolytic side activities. However, upon addition of Asc an autooxidation of the TXG could be observed, resulting in a visible decrease in the MW distribution after 24 h (Additional file 1: Fig. S1). Therefore, the MW distributions of the *Nc*LPMO digests (with Asc) were compared to the ones of TXG without enzyme but with Asc (24 h; Fig. 2).

Already after 2 h, the products formed by *Nc*LPMO9M had a lower MW-range compared to *Nc*LPMO9C indicating that both LPMOs show distinct mode-of-action on TXG (Fig. 2). To be more precise, *Nc*LPMO9M formed two rather broad populations (Fig. 2a), one ranging from 30–200 kDa and another ranging from 1–30 kDa, while *Nc*LPMO9C formed two larger MW populations (80–700 kDa and 1–80 kDa, Fig. 2b). Notably, oxidative XG cleavage of *Nc*LPMO9M has not been reported previously and neither have the MW distributions of XG digests of these *Nc*LPMOs. Further, seen from the MW profiles (Fig. 2a), after 8 h the *Nc*LPMO9M TXG degradation was complete, no high MW population (30–200 kDa) of XG remained, and final products ranged from 0.4 to 3 kDa (Fig. 2a). In contrast, for the *Nc*LPMO9C-TXG-digest the high MW XG population (80–700 kDa) remained and a decrease in MW of the products was observed even between 8 h and 24 h of incubation (Fig. 2b). The final digest was composed of products ranging from 0.4 to 3 kDa and showed a different MW distribution profile as the 24 h *Nc*LPMO9M-TXG-digest (Fig. 2b).

**Fig. 3:**
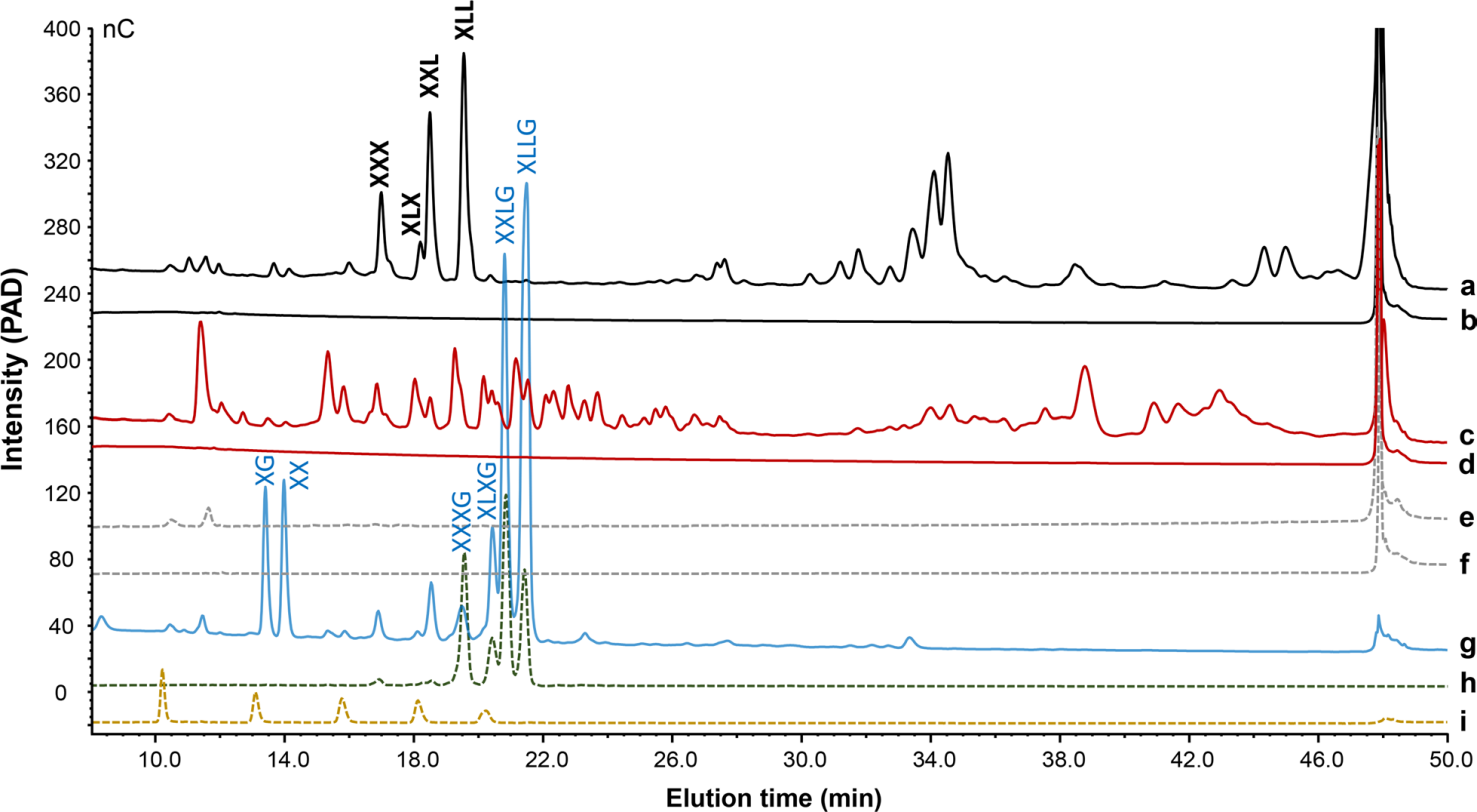
HPAEC elution patterns of oligosaccharide products after the incubation of tamarind seed xyloglucan (TXG) with *Nc*LPMO9C (1.25 μM; a, b) and *Nc*LPMO9M (1.25 μM; c, d) in the presence (1 mM; a, c) and absence (b, d) of ascorbic acid (Asc). TXG oligosaccharides released by xyloglucanase (XEG, 1.25 μM) in the presence of Asc (g) were added as the reference. In addition, TXG only (f), TXG with 1 mM Asc (e), TXG oligosaccharide standards (xyloglucan hepta + octa + nona saccharides; h) and a standard (i) containing a mixture of cellobiose, cellotriose, cellotetraose, cellopentaose and cellohexaose (from left to right in chromatogram) are shown

To learn more about the exact cleavage sites in the TXG for both *Nc*LPMOs, the formed TXG oligosaccharides were characterized in detail. First, the digests were analyzed by HPAEC-PAD and the corresponding chromatograms are shown in Fig. 3. For comparison, the commercial xyloglucanase (XEG)-TXG-digest (Fig. 3g) and commercial non-oxidized TXG oligosaccharide (XXXG, XLXG, XXLG and XLLG) standards (Fig. 3h) were analyzed, of which the annotation of HPAEC-peaks has been well defined in literature [42–44]. The control reactions (Fig. 3b, d) did not show the formation of (detectable) oligosaccharides, which confirms the absence of hydrolytic xyloglucanase (side-)activities. In the presence of Asc, both *Nc*LPMOs released noticeably different types of TXG oligosaccharides (Fig. 3a, c), underlining the differences in the above-described MW distributions (Fig. 2). The TXG-digest of *Nc*LPMO9C has been described previously and our HPAEC profile corresponds with the published one [32]. However, the annotation, of in particular the non-oxidized products, seems to be different compared to the previous research. Based on our results, the common non-oxidized “XXXG”-type products were not present in *Nc*LPMO9C-TXG-digest (Fig. 3a). Our annotation was based on (i) comparison with the XEG-TXG-digest and standards of a mixture of XXXG, XLXG, XXLG and XLLG (Fig. 3g, h), and (ii) β-galactosidase treatment of the *Nc*LPMO9C- and XEG-TXG-digest to confirm that L units were degraded to X units (Additional file 1: Fig. S2). Indeed, β-galactosidase treatment of the XEG-TXG-digest (Additional file 1: Fig. S2b) resulted in removal of XLXG, XXLG and XLLG, and only XXXG remained. In addition, XXG was formed, confirmed by MALDI-TOF-MS (Additional file 1: Fig. S3a; *m/z* 775.3 (lithium (Li)-adduct, [M+Li]^+^)), due to the presence of isoprimeverase in the commercial β-galactosidase [45, 46], which was further substantiated by the formation of isoprimeverose (X unit) (Additional file 1: Fig. S2b). In contrast, β-galactosidase treated *Nc*LPMO9C-TXG-digest, majorly resulted in XXX (Additional file 1: Fig. S2d), which was confirmed by MALDI-TOF-MS (Additional file 1: Fig. S3b; *m/z* 907.3 ([M+Li]^+^)), and no other main non-oxidized compounds remained. Again minor isoprimeverase side-activity was seen, resulting in formation of X and XX. The peak representing XXX was also present in the *Nc*LPMO9C-TXG-digest, without β-galactosidase treatment, in addition to three peaks now defined as XLX, XXL and XLL. These last three peaks were removed by the β-galactosidase treatment, which confirmed the presence of L unit. It should be noted that in previous research studying LPMO activity towards TXG, the non-oxidized oligosaccharides now annotated as XXX, XLX, XXL and XLL, were incorrectly suggested to be XXXG, in addition to XLXG, XXLG and XLLG [22, 24, 29, 31, 32].

The HPAEC pattern of the *Nc*LPMO9M-TXG-digest showed considerably more oligosaccharides peaks compared to the TXG-digest of *Nc*LPMO9C. As with HPAEC the type of oligosaccharides (especially the oxidized ones) formed cannot be identified without standards, further characterization of degraded TXG oligosaccharides was carried out by MALDI-TOF-MS and HILIC-ESI-CID-MS/MS.

**Fig. 4:**
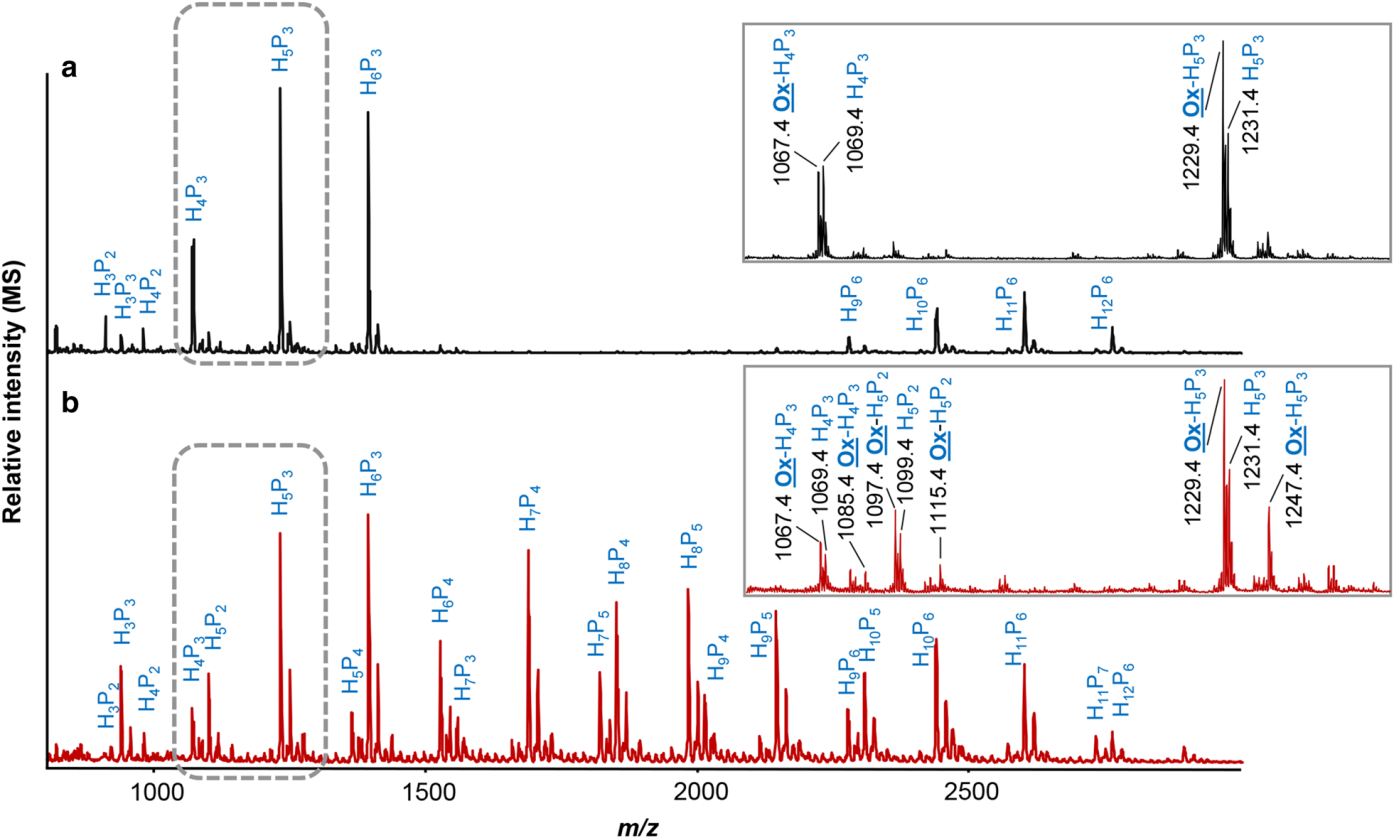
MALDI-TOF mass spectra of tamarind seed xyloglucan (TXG), after incubation with **a** 1.25 µM *Nc*LPMO9C and **b** 1.25 µM *Nc*LPMO9M, both in the presence of 1 mM of ascorbic acid (Asc). TXG oligosaccharide clusters included non-oxidized oligosaccharides (H_n_P_m_) and C4-oxidized oligosaccharides (Ox-H_n_P_m_). The clusters of H_4_P_3_ and H_5_P_3_ are enlarged in the inserts. Abbreviations: H, hexaose (glucose or galactose, 162 Da); P, pentaose (xylose, 132 Da); n, number of hexaoses; m, number of pentaoses; Ox, oxidized. *M/z* shown correspond to lithium (Li)-adducts

The MALDI-TOF mass spectrum of the *Nc*LPMO9C-TXG-digest (Fig. 4a) clearly indicated masses ([M+Li]^+^) corresponding to blocks of TXG, which has also been shown in the previous research [32, 46]. These blocks were present as non-oxidized (i.e., H_4_P_3_, H_5_P_3_ and H_10_P_6_) and C4-oxidized oligosaccharides (i.e., Ox-H_4_P_3_ and Ox-H_5_P_3_), where “H” and “P” represented as hexaose and pentaose, respectively. Taking the above-described HPAEC results into account, it could be concluded that, for instance, H_4_P_3_ represents XXL/XLX, and H_5_P_3_ represents XLL. The annotation of C4-oxidized XG oligosaccharides (i.e., Ox-H_4_P_3_ (*m/z* 1067.4)) was based on the − 2 *m/z* difference compared to the *m/z*-value of the corresponding non-oxidized block (i.e., H_4_P_3_ (*m/z* 1069.4; Fig. 4a) and is comparable to previous annotations of C4-oxidized LPMO products [23, 30, 47]. The C4-oxidized TXG oligosaccharides were determined to be of “XXXG”-type, as in the β-galactosidase treated *Nc*LPMO9C-TXG-digest analyzed by MALDI-TOF-MS, a major peak with *m/z* 1067.4 ([M+Li]^+^), representing C4-oxidized XXXG, remained (Additional file 1: Fig. S3b). The C4-selectivity of *Nc*LPMO9C towards TXG previously has been reported by Agger and coworkers [32].

The *Nc*LPMO9M-TXG-digest showed *m/z*-values ([M+Li]^+^) corresponding to many different types of TXG oligosaccharides (i.e., H_7_P_5_; Fig. 4b). The *Nc*LPMO9M-TXG-digest was again composed of both non-oxidized (i.e., H_5_P_3_ (*m/z* 1231.4) and oxidized oligosaccharides (i.e., Ox-H_5_P_3_; *m/z* 1229.4 and 1247.4; Fig. 4b). The *m/z* difference of +16 suggested the occurrence of C1-oxidation and can be explained by the spontaneous hydrolysis of the unstable δ-lactone form (− 2 Da) into the aldonic acid form (+ 16 Da) [23, 30, 47]. Although some studies have shown that *m/z* of +16 could also attribute to the gem-diol form of the C4-oxidized products, other studies, i.e., in our laboratory, by using the same MALDI-TOF-MS settings as in the current work, did not observe *m/z* of +16 for C4-oxidized products [30, 47]. Therefore, we suggest that TXG, most likely, was oxidatively cleaved by *Nc*LPMO9M at C1 position. Still, occurrence of C4-oxidation could not be excluded, because of the presence of oxidized oligosaccharides with the *m/z* difference of − 2. These masses (M − 2) not only represent the unstable δ-lactone form, but also the keto-form of C4-oxidized oligosaccharides [30, 32, 48].

### Unambiguous structural characterization of XG degradation products generated by *Nc*LPMO9C and *Nc*LPMO9M

**Fig. 5:**
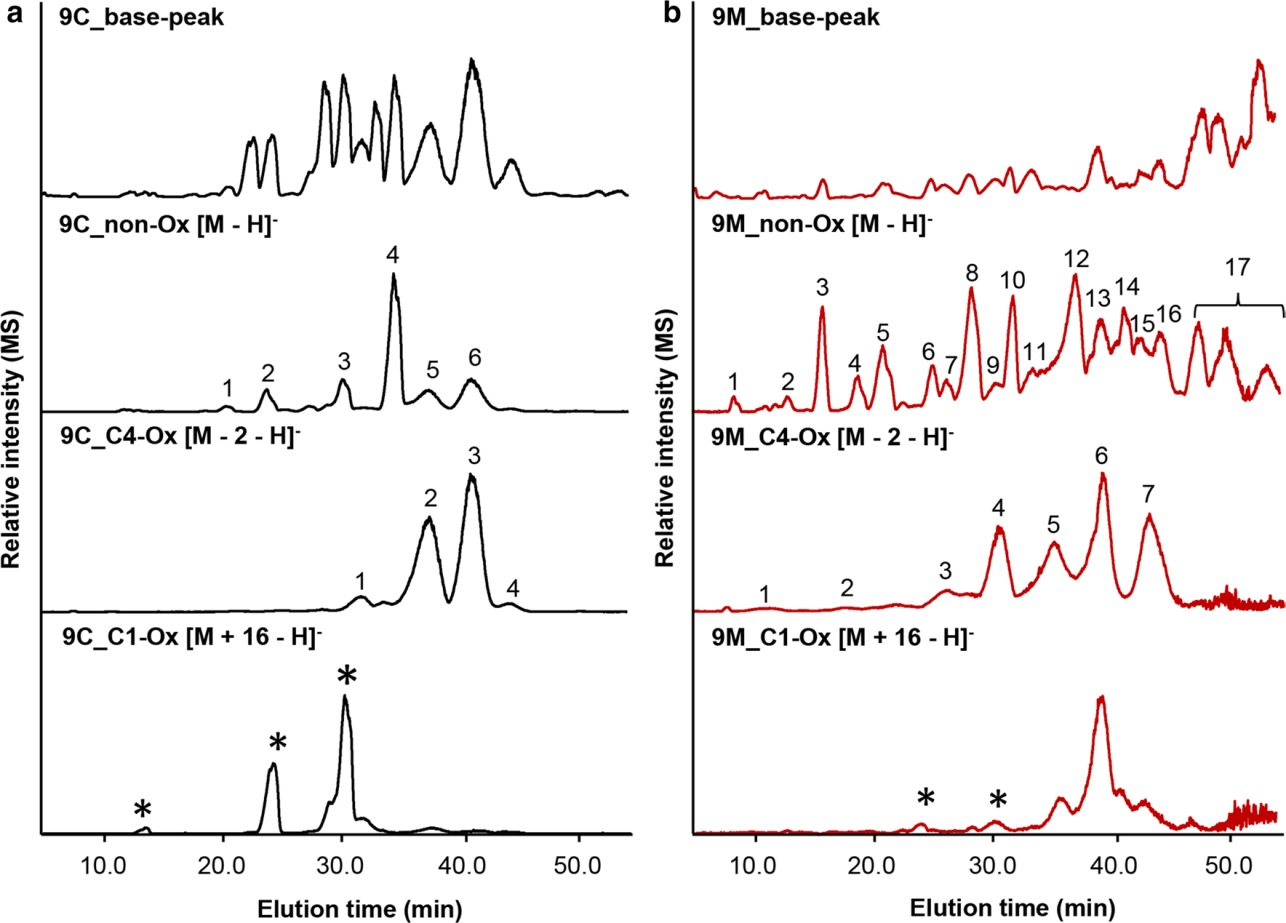
HILIC-ESI-MS base-peak and extracted ion chromatograms of tamarind seed xyloglucan (TXG) digests from **a***Nc*LPMO9C and **b***Nc*LPMO9M. Extracted ion chromatograms are made for non-, C4- and C1-oxidized products (non-Ox, C4-Ox and C1-Ox) released by *Nc*LPMO9C and *Nc*LPMO9M (in the presence of 1 mM ascorbic acid). Determination of C4- and C1-oxidized TXG oligosaccharides is based on the *m/z* difference of − 2 and + 16, respectively, compared to *m/z*-values of corresponding non-oxidized oligosaccharides. The identification (numbered peaks) of C4-oxidized TXG oligosaccharides in *Nc*LPMO9C- and *Nc*LPMO9M-TXG-digest, based on the MS/MS fragmentation patterns, is shown in Tables 2 and 3, respectively. Asterisks indicate formic acid adducts of non-oxidized TXG oligosaccharides having the same *m/z* as C1-oxidized products

To further identify the exact TXG cleavage sites of the two *Nc*LPMOs, digests were subjected to negative ion mode HILIC-ESI-CID-MS/MS. Similar to the data discussed above (Figs. 2, 3, 4), the HILIC-ESI-MS patterns of the two LPMO-TXG-digests were different (Fig. 5). Firstly, the masses in the base-peak chromatograms of both digests showed that non-oxidized TXG oligosaccharides were present as single charged deprotonated ([M−H]^−^) and double charged deprotonated ([M−2H]^2−^) products (data not shown, M indicates the *m/z* of non-oxidized oligosaccharides). The same was observed for oxidized oligosaccharides represented by the *m/z* − 2 products ([M−2−H]^−^, [M−2−2H]^2−^) and the *m/z* + 16 products ([M+16−H]^−^, [M+16−2H]^2−^) compared to the same degree of polymerization (DP) of non-oxidized oligosaccharides. Secondly, masses that could be either C1-oxidized products or, based on their mass, formic acid adducts of non-oxidized products were observed (Fig. 5). For instance, *m/z* 1107 could represent the C1-oxidized H_5_P_2_, but also the formic acid adduct of non-oxidized H_4_P_3_ (Additional file 1: Table S1). Nevertheless, corresponding MS/MS data easily distinguished formic acid adducts as these products showed a clear fragment of *m/z* − 46 (formic acid; data not shown).

**Fig. 6:**
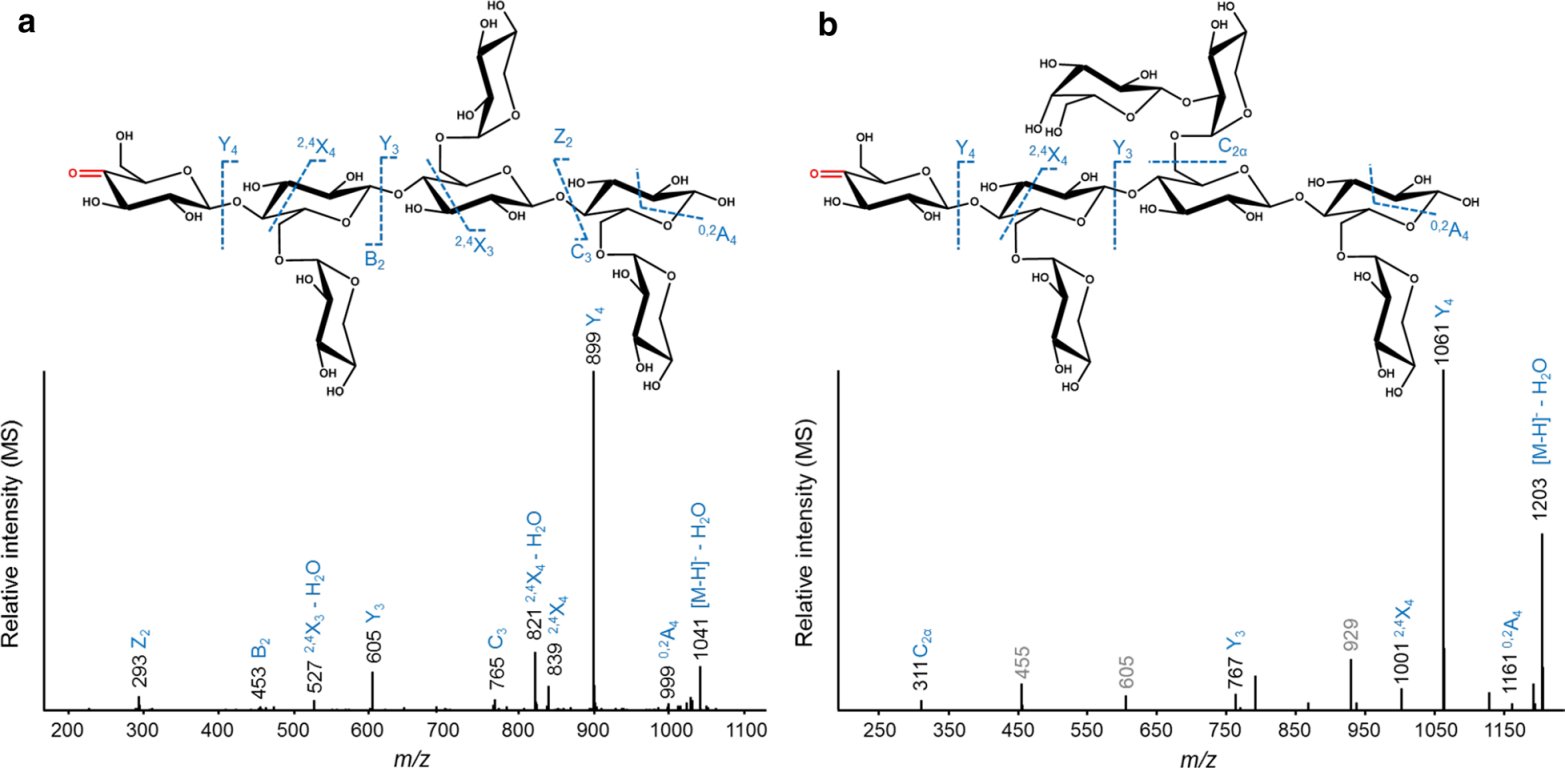
Negative ion mode CID-MS/MS fragmentation patterns of C4-oxidized tamarind seed xyloglucan (TXG) oligosaccharides present in the *Nc*LPMO9C-TXG-digest annotated as _O=G_GXXX (*m/z* 1059.4, **a**) and _O=G_GXLX (*m/z* 1221.5, **b**). _O=G_ indicates that the oxidation is on the glucosyl unit in keto-form. Oxidation of the C4-carbon position is indicated in red. The fragments are annotated according to the nomenclature proposed by Domon and Costello [49]. The *m/z*-values shown in grey in b are from the other co-eluted isomers

**Fig. 7:**
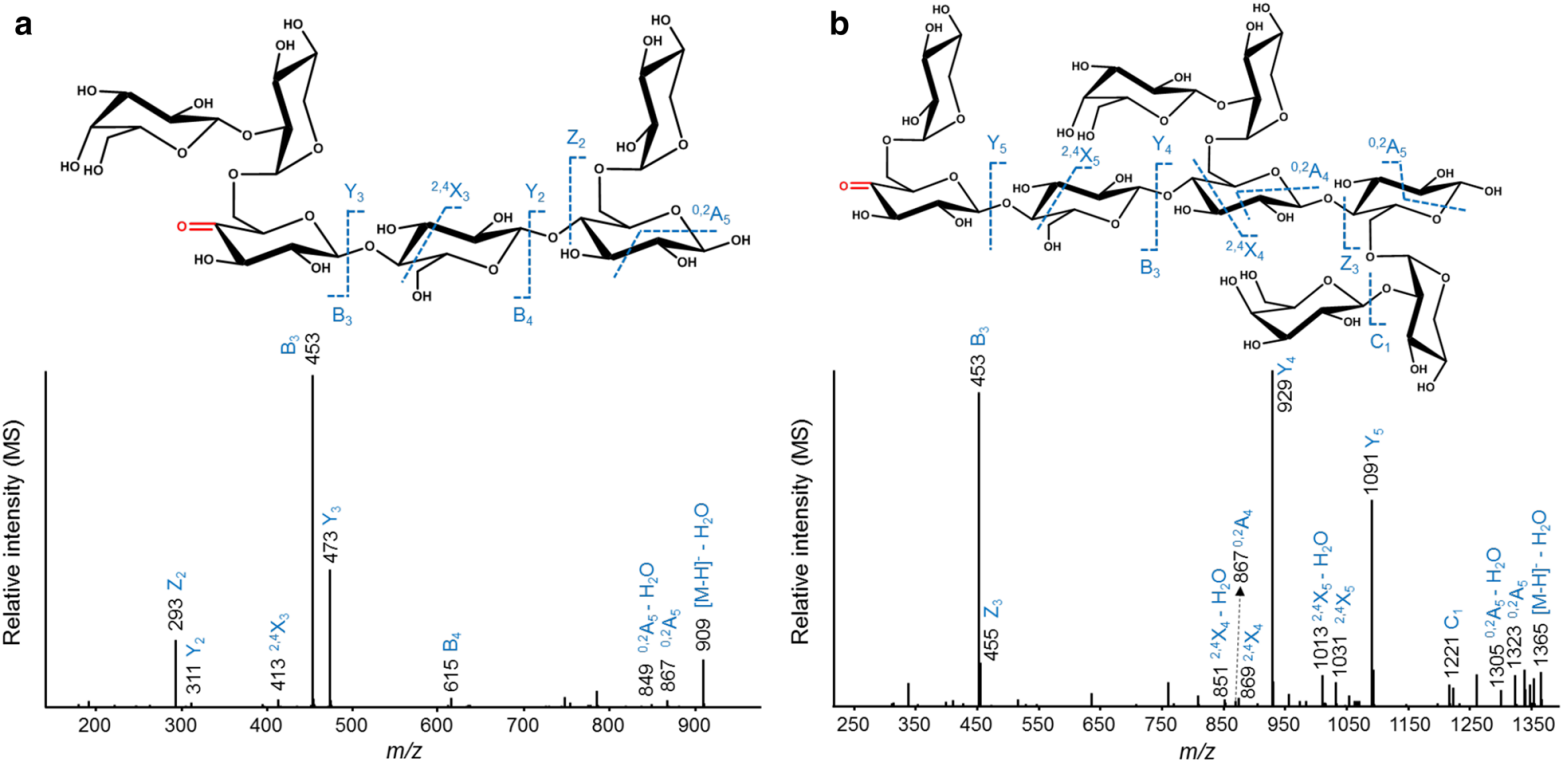
Negative ion mode CID-MS/MS fragmentation patterns of C4-oxidized tamarind seed xyloglucan (TXG) oligosaccharides present in the *Nc*LPMO9M-TXG-digest annotated as _O=G_LGX (*m/z* 927.3, **a**) and _O=G_XGLL (*m/z* 1383.7, **b**). _O=G_ indicates that the oxidation is on the glucosyl unit in keto-form. Oxidation of the C4-carbon position is indicated in red. The fragments are annotated according to the nomenclature proposed by Domon and Costello [49]

Due to the complexity of multiple charges and formic acid adducts, the intensity of the MS/MS spectra was too poor for structural elucidation. The spectral quality improved considerably after having established MS and MS/MS analysis via a defined mass list (Additional file 1: Table S1; [M−H]^−^, [M−2−H]^−^, [M+16−H]^−^). The chromatograms and spectra obtained via the mass list allowed structural characterization of the non-oxidized and oxidized TXG oligosaccharides released by *Nc*LPMO9C and *Nc*LPMO9M (Figs. 5, 6, 7, Tables 2, 3).

**Table 2:** List of C4-oxidized XG oligosaccharides identified based on fragmentation patterns in CID-MS/MS present in the *Nc*LPMO9C-TXG-digest

9C_C4-Ox Peak Nr.	Elution time (min)	*m/z* ([M−H]^−^)	MS/MS fragments (*m/z*)^a^	Annotation
1	30.7–32.7	1059.4	*293 (4)*, *453 (1)*, 527 (3), *605 (13)*, *765 (1)*, *821 (16)*, *839 (7)*, *899 (100)*, 999 (2), 1041 (14)	_O=G_GXXX
			*455 (1)*, *473 (1)*, *767 (4)*, 999 (2), 1041 (14)	_O=G_XXXG
2	36.4–37.5	1221.5	311 (2), *767 (4)*, *1001 (5)*, *1061 (100)*, 1161 (2), 1203 (34)	_O=G_GXLX/_O=G_GXXL
			311 (2), *605 (3)*, *1001 (5)*, *1061 (100)*, 1161 (2), 1203 (34)	_O=G_GLXX
			455 (8), *929 (12)*, 1161 (2), 1203 (39)	_O=G_X(H_4_P_2_)
3	39.7–41.4	1383.7	311 (3), *1141 (1)*, *1163 (5)*, *1223 (100)*, 1365 (7)	_O=G_G(H_5_P_3_)
4	43.0–45.0	1515.5	n.d.	_O=G_H_6_P_4_

Chromatograms, including peak numbers, are shown in Fig. 5. Nomenclature (annotation) according to Fry et al. [15]

^a^Relative intensities of MS/MS fragments are shown between brackets and informative MS/MS fragments are indicated in italics

**Table 3:** List of C4-oxidized XG-oligosaccharides identified based on fragmentation patterns in CID-MS/MS present in the *Nc*LPMO9M-TXG-digest

9M_C4-Ox Peak Nr.	Elution time (min)	*m/z* ([M−H]^−^)	MS/MS fragments (*m/z*)^a^	Annotation
1	10.7–11.7	471.2	*159 (59)*, 293 (15), 311 (15), 353 (6), 395 (14), 453 (20)	_O=G_GX
			179 (20), *291 (2)*, 351 (3), 221 (3), 395 (14), 453 (20)	_O=G_XG
2	17.5–18.7	765.4	*291 (100)*, 473 (3), 543 (1), *603 (1)*, 747 (9)	_O=G_XXG
			*291 (100)*, 293 (1), 353 (1), *453 (1)*, 455 (1), 471 (1), 473 (3), 747 (9)	_O=G_XGX
3	24.1–27.9	927.3	*291 (1)*, 353 (1), *411 (1)*, *453 (100)*, 455 (1), 473 (18), *635 (4)*, 747 (1), *765 (1)*, 867 (2), 909 (3)	_O=G_XGL
			*293 (8),* 353 (1), *707 (1)*, *767 (1),* 867 (2), 909 (3)	_O=G_GLX
4	27.9–32.0	927.3	*293 (20)*, *311 (1)*, *413 (2)*, *453 (100)*, 473 (42), *615 (3)*, 747 (2), 849 (1), 867 (3), 909 (13)	_O=G_LGX
	30.5–31.5	1059.4	*291 (37)*, *293 (3)*, *353 (4)*, *455 (33)*, *473 (2)*, *707 (18)*, *765 (4)*, *767 (66)*, 999 (1), 1041 (100)	_O=G_XXGX
			*291 (37)*, *293 3), 353 (4)*, *605 (7)*, *526 (3), 765 (4)*, *767 (66)*, 999 (1), 1041 (100)	_O=G_XGXX
5	32.5–34.1	1089.1	*453 (100)*, *557 (3)*, *635 (28), 867 (5)*, *927 (7)*, 1029 (6), 1071 (33)	_O=G_LLG
			*453 (100)*, *473 (12)*, *557 (5)*, *635 (28),* 1029 (6), 1071 (33)	_O=G_LGL
	33.9–34.9	1221.5	*453 (100)*, *455 (4)*, 689 (2), *767 (32)*, *869 (4)*, *929 (15)*, 1161 (5), 1203 (5)	_O=G_XGXL
			*293 (3)*, 689 (2), *767 (32)*, *869 4)*, *929 (15)*, 1161 (5), 1203 (5)	_O=G_XGLX
			*293 (3)*, *453 (100)*, *473 (8)*, 689 (2), *767 (32)*, 1161 (5), 1203 (5)	_O=G_LXGX
6	38.1–38.9	1221.5	353 (3), *453 (100)*, *455 (18)*, 689 (7), 707 (13), *767 (56)*, *929 (52)*, 1161 (2), 1203 (92)	_O=G_XGXL
			*453 (100)*, *473 (7)*, 689 (7), 707 (13), *767 (56)*, 1161 (2), 1203 (92)	_O=G_LXGX
	38.6–39.6	1383.7	*411 (2), 453 (96*), 515 (2), *851 (2)*, *867 (2)*, *869 (2)*, *929 (100)*, *1013 (11)*, *1031 (8)*, *1091 (71)*, 1221 (10), 1305 (6), 1323 (8), 1365 (9)	_O=G_XGLL
7	41.7–44.4	1383.7	453 (13), 455 (4), 635 (3), 767 (1), 851 (3), 869 (3), 929 (100), 1091 (2), 1223 (2), 1365 (17)	_O=G_H_6_P_3_
		1515.5	n.d.	_O=G_H_6_P_4_

Chromatograms, including peak numbers, are shown in Fig. 5. Nomenclature (annotation) according to Fry et al. [15]

^a^Relative intensities of MS/MS fragments are shown between brackets and informative MS/MS fragments are indicated in italics

#### Characterization of non-oxidized TXG oligosaccharide products

Multiple non-oxidized TXG oligosaccharides released by the two *Nc*LPMOs were identified (see Additional file 1: Figs. S4, S5 for examples). A summary of all MS/MS fragments and structural annotations can be found in Additional file 1: Table S2 (for *Nc*LPMO9C) and Additional file 1: Table S3 (for *Nc*LPMO9M). MS/MS fragments of non-oxidized products were annotated following the principle of predominance of C/Z-type and A-type fragments of neutral oligosaccharides in negative MS-mode [49, 50]. In addition, a double C/Z-type cleavage on three linked sugar residues was observed and annotated as D-type (Additional file 1: Figs. S4, S5), which has previously been reported for TXG oligosaccharides [50]. Overall, non-oxidized XXX (*m/z* 899.3, Additional file 1: Fig. S4a), XXL (*m/z* 1061.4), XLX (*m/z* 1061.4), XLL (*m/z* 1223.5, Additional file 1: Fig. S4b) and GXLL (*m/z* 1385.7) were formed in the *Nc*LPMO9C-TXG-digest (Additional file 1: Table S2). These non-oxidized “XXX”-type TXG oligosaccharides reflected cleavage at the non-reducing end of an unbranched glucosyl unit in TXG (see below). In summary, 19 different non-oxidized TXG oligosaccharides released by *Nc*LPMO9M were identified (Additional file 1: Tables S3, Fig. S5).

#### Characterization of C4-oxidized TXG oligosaccharide products

Based on our previous study on CID-MS/MS fragmentation patterns of C4-oxidized cello-oligosaccharides [51], we identified multiple structures of C4-oxidized TXG oligosaccharides, which are shown in Tables 2 and 3, for *Nc*LPMO9C and *Nc*LPMO9M, respectively. In the *Nc*LPMO9C-TXG-digest, we found several “XXXG”-type C4-oxidized products such as _O=G_GXXX (*m/z* 1059.4, _O=G_ indicates the C4-oxidized glucosyl unit), _O=G_GXLX (*m/z* 1221.5), _O=G_GXXL (*m/z* 1221.5), _O=G_GLXX (*m/z* 1221.5) and _O=G_G(H_5_P_3_) (*m/z* 1383.7) (Table 2). To explain the identification of these compounds, for instance through annotation of MS/MS fragments of _O=G_GXXX (*m/z* 1059.4, Fig. 6a) and _O=G_GXLX (*m/z* 1221.5, Fig. 6b), a fragment (Y_4_) was observed having the terminal oxidized unbranched glucosyl residue removed via B/Y-cleavage (*m/z* difference of 160 compared to the parent *m/z*). In addition, the diagnostic cross-ring fragment ^2,4^X_4_ confirmed the single C4-oxidation on an unbranched glucosyl unit. This diagnostic cleavage fragment has been shown for C4-oxidized cello-oligosaccharides as well [51]. Additionally, to a much lesser extent, oligosaccharides with a C4-oxidized terminal X unit were determined, such as in _O=G_XXXG (*m/z* 1059.4) and _O=G_X(H_4_P_2_) (*m/z* 1221.5). Again, fragments resulting from B/Y-cleavage of the glycosidic linkage between the glucosyl units next to the C4-oxidized glucosyl unit were observed in the MS/MS spectra. Fragments of (*m/z*) 767 and 929 showed a 292 *m/z* difference compared to the parent *m/z* of 1059 and 1221, respectively. The 292 *m/z* difference indicated the loss of the oxidized glucosyl unit (*m/z* 160) substituted with a xylosyl residue (*m/z* 132).

C4-oxidized TXG oligosaccharides released by *Nc*LPMO9M were different from the ones formed by *Nc*LPMO9C, which is summarized in Table 3. First, two small motifs, _O=G_GX and _O=G_XG (both *m/z* 471.2), were identified. The single C4-oxidation on these G and X units was confirmed by MS/MS fragments of *m/z* 159 and 291, respectively. In addition, C4-oxidized oligosaccharides not having “XXXG”-type structure were detected mainly including _O=G_XXG (*m/z* 765.4), _O=G_XGX (*m/z* 765.4), _O=G_XGL (*m/z* 927.3), _O=G_GLX (*m/z* 927.3), _O=G_LGX (*m/z* 927.3). Other structures such as _O=G_LLG (*m/z* 1089.1) and _O=G_LGL (*m/z* 1089.1) were also identified (Table 3). Among these structures, the single C4-oxidation of G and X units was elucidated by MS/MS fragments having *m/z* differences of 160 and 292 from their parent *m/z*, respectively, as described previously. An example for the identification of _O=G_L units in MS/MS is shown in Fig. 7a, where the B_3_ (*m/z* 453) indicated the oxidation on the H_2_P_1_ structure (_O=G_H_2_P_1_). However, _O=G_H_2_P_1_ has three isomeric structures: _O=G_L, _O=G_XG and _O=G_GX. These three structures were further distinguished by the ion B_4_ (_O=G_H_3_P_1_, *m/z* 615) and the cross-ring fragment ^2,4^X_3_ (an X unit and a cross-ring cleaved G unit, *m/z* 413). Altogether, including the *m/z* of the parent oligosaccharide (_O=G_H_4_P_2_, *m/z* 927.3), it is concluded that _O=G_LGX represented *m/z* 927.3.

All above-mentioned motifs were generated by the oxidative XG cleavage of *Nc*LPMO9M at the non-reducing end of substituted glucosyl units from “XXXG”-type building block of TXG. Furthermore, the C4-oxidized oligosaccharides having an *m/z*-value of 1059.5 (_O=G_H_4_P_3_) in *Nc*LPMO9M-TXG-digest were composed of mainly _O=G_XXGX and _O=G_XGXX instead of compounds having terminal G units (for example _O=G_GXXX and _O=G_XXXG in the *Nc*LPMO9C-TXG-digest). Similarly, an *m/z*-value of 1221.5 was also annotated as mainly _O=G_XGXL, _O=G_XGLX and _O=G_LXGX and an *m/z*-value of 1383.7 was _O=G_XGLL (only one was identified, Fig. 7b) in the *Nc*LPMO9M-TXG-digest.

#### Characterization of C1-oxidized TXG oligosaccharide products

C1-oxidized products were only detected in the *Nc*LPMO9M-TXG-digest. However, due to the poor signal intensity and heavy co-elution of all C1-oxidized products in HILIC-ESI-MS, these products could not be structurally identified. Nevertheless, the presence of the parent masses of C1-oxidized products confirmed that *Nc*LPMO9M resulted in both C1- and C4-oxidized XG oligosaccharides.

#### Characterization of (oxidized) BCXG oligosaccharide products

We further analyzed the cleavage patterns of *Nc*LPMO9C- and *Nc*LPMO9M-digests towards BCXG which is a XG having additional F units (glucosyl-xylosyl-galactosyl-fucosyl residue; Table 1), again by using HILIC-ESI-CID-MS/MS (Additional file 1: Fig. S6). The HILIC-ESI-MS base-peak chromatograms of two *Nc*LPMO-BCXG-digests showed once more the striking difference between the patterns (Additional file 1: Fig. S6a, b). Due to the high complexity, not all released (oxidized) BCXG degradation products by LPMOs were fully elucidated. Nevertheless, in the *Nc*LPMO9C-BCXG-digest, we were able to identify BCXG oligosaccharides with a C4-oxidized terminal G unit (e.g., _O=G_GXXF, *m/z* 1367.7, Additional file 1: Fig. S6c), which is absent in the *Nc*LPMO9M-BCXG-digest. Interestingly, a diagnostic C4-oxidized F unit (_O=G_F(H_3_P_2_), *m/z* 1367.7, Additional file 1: Fig. S6d) was identified in the *Nc*LPMO9M-BCXG-digest, which was absent in the *Nc*LPMO9C-BCXG-digest. The identified C4-oxidized F unit indicated that oxidative cleavage of BCXG by *Nc*LPMO9M also occurred next to the extensively substituted glucosyl units.

#### Distinct mode-of-action of NcLPMO9C and NcLPMO9M towards XG

**Fig. 8:**
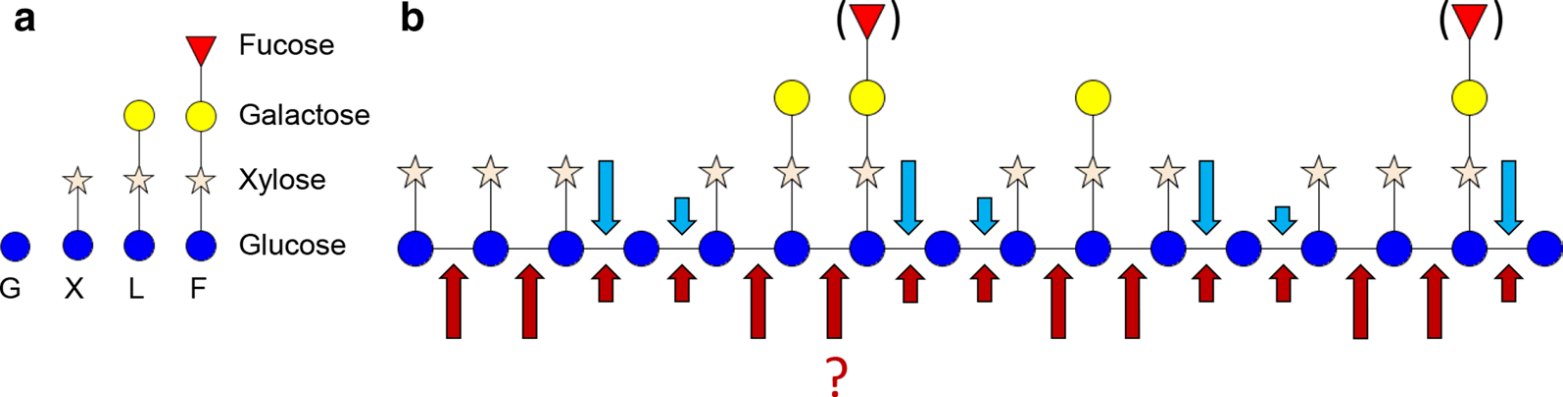
**a** Illustration of structural units in xyloglucan (XG) and **b** schematic representation of XG cleavage patterns by *Nc*LPMO9C (blue arrows) and *Nc*LPMO9M (red arrows), respectively. G unit, glucosyl residues only (blue circle); X unit, glucosyl-xylosyl residues (xylose, star); L unit, glucosyl-xylosyl-galactosyl residues (galactose, yellow circle) and F unit, glucosyl-xylosyl-galactosyl-fucosyl residues (fucose, red triangle). Positions of galactosyl units may vary and fucosyl units are present in black currant XG, but not in tamarind seed XG. *Nc*LPMO9C showed substitution-intolerant mode-of-action meaning that its oxidative cleavage towards XG was (predominately) at the non-reducing end of unbranched G units, while *Nc*LPMO9M oxidatively cleaved XG regardless of substitution (substitution-tolerant) with seemingly preference on substituted glucosyl units. Whether *Nc*LPMO9M can cleave between two L units remains to be studied and is shown as red question mark. The size of the arrows is indicative for more pronounced cleavage sites, which was based on (the number of) structures found of identifiable (oxidized) oligosaccharides by using HILIC-ESI-MS

In this study, the structures of oxidized TXG oligosaccharides generated by two *Nc*LPMO9C (Table 2) and *Nc*LPMO9M (Table 3) from XG were unambiguously elucidated. In the *Nc*LPMO9C-TXG-digest, TXG oligosaccharides were found mostly to be typical “XXXG”-type block units, but with C4-oxidized unbranched G units (e.g., _O=G_GXXX, _O=G_GXLX, _O=G_GXXL, _O=G_GLXX and _O=G_G(H_5_P_3_)). Another C4-oxidized “XXXG”-type product (_O=G_GXXF) was identified in the *Nc*LPMO9C-BCXG-digest. In contrast, non-“XXXG”-type of C4-oxidized TXG oligosaccharides were identified in the *Nc*LPMO9M-TXG-digest. The C4-oxidation of TXG oligosaccharides by *Nc*LPMO9M on X and L units confirmed that *Nc*LPMO9M can oxidize substituted glucosyl units at the C4-carbon. In addition, the oxidation predominately found on X and L units in HILIC-ESI-CID-MS/MS characterized TXG oligosaccharides, instead of on unbranched G units, may reflect that *Nc*LPMO9M has the preference in cleaving the substituted glucosyl backbone. The identified C4-oxidized F unit from *Nc*LPMO9M-BCXG-digest further indicated that the oxidative cleavage of XG by *Nc*LPMO9M is independent of the type and length of the branches. Based on these determined XG cleavage sites, it was defined that *Nc*LPMO9C oxidatively cleaves XG predominantly at the non-reducing end of single unbranched glucosyl units [32], further referred to as a substitution-intolerant mode-of-action towards XG (in brief “Substitution-intolerant”) (Fig. 8). In contrast, the oxidative cleavage of XG by *Nc*LPMO9M was shown to be more tolerant to substitutions with even a preference next to substituted glucosyl units and referred to as “Substitution-tolerant” (Fig. 8).

### Phylogenetic and structural analysis of LPMOs with XG activity

**Fig. 9:**
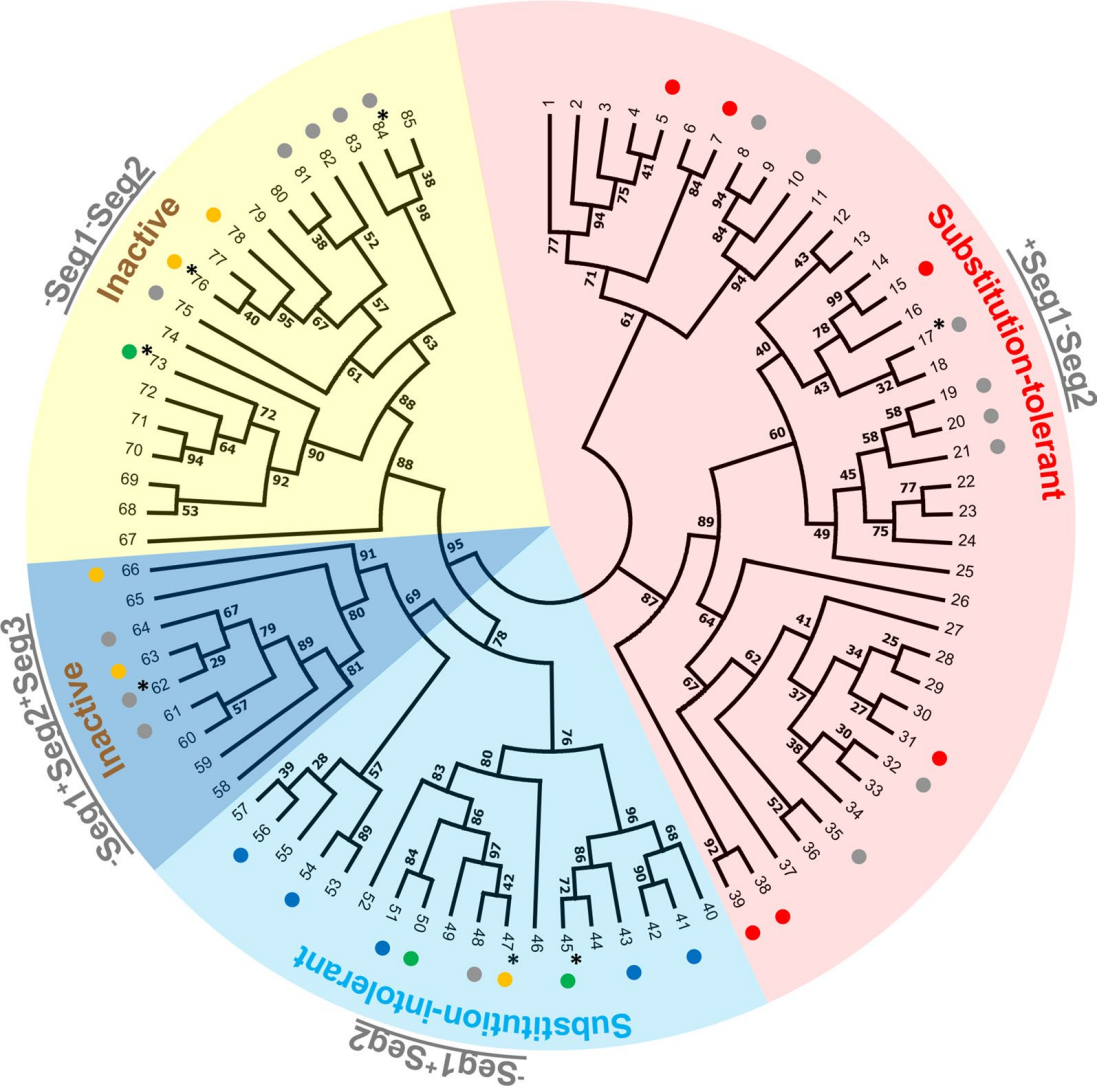
Unrooted topology tree based on active site segments only of AA9 LPMOs with numbering on the terminal nodes. Corresponding organism name, Genbank accession number, LPMO name (if characterized) and PDB entry (if applicable) of each number in the terminal node is listed in Additional file 1: Table S4. Background colors in the topology tree show the long (^+^)/short (^−^) of Seg1 and Seg2 segments [red, ^+^Seg1^−^Seg2; light blue, ^−^Seg1^+^Seg2; dark blue, ^−^Seg1^+^Seg2 but extended Seg3 (^+^Seg3); yellow, ^−^Seg1^−^Seg2]. Reported characterized LPMOs are indicated by colored dots; XG product patterns alike that of *Nc*LPMO9C (blue, Substitution-intolerant), alike that of *Nc*LPMO9M (red, Substitution-tolerant) or not XG-active (yellow, Inactive). Grey dots indicate that the LPMOs were reported for 1) their activity on cellulose only and not tested on XG (e.g., No. 10, *Hi*LPMO9B) or 2) their cellulolytic enhancing activity and not tested on XG (e.g., No. 35, *Af*AA9_B) or 3) tested with XG but the data were not conclusive (e.g., No. 20, *Pa*LPMO9D). Green dots indicate that oxidative XG cleavage has been shown, but reported data are inconclusive to be determined as substitution-intolerant or -tolerant. LPMOs with red dots: 5. *Nc*LPMO9M; 7. *Gt*LPMO9A-2; 15. *Fg*LPMO9A; 31. *Ta*LPMO9A; 38. *Gc*LPMO9B; 39. *Gc*LPMO9A. LPMOs with blue dots: 41. *Mt*LPMO9J; 43. *Nc*LPMO9C; 51. *Nc*LPMO9D; 54. *Cv*AA9A; 56. *Ls*AA9A. LPMOs with yellow dots: 47. *Nc*LPMO9A; 63. *Mt*LPMO9B; 66. *Mt*LPMO9I; 76. *Tt*LPMO9E; 78. *Nc*LPMO9F. *17. *Pa*LPMO9D: XG catalytic performance was determined based on a colorimetric H_2_O_2_-production assay [31]. *45. *Pa*LPMO9H: XG product profiles were shown to be either *Nc*LPMO9C-like (“Substitution-intolerant”) via HPAEC [31] and *Nc*LPMO9M-like (“Substitution-tolerant”) via direct infusion ESI-MS/MS [26], hence inconclusive. *47. *Nc*LPMO9A: “Inactive” on XG alone but “Substitution-intolerant” in combination with cellulose [20]. *62. *Pa*LPMO9B: XG catalytic performance was determined based on a colorimetric H_2_O_2_-production assay [31]. *73. AN3046: Only one XG product profile was shown (MALDI-TOF mass spectrum), hence inconclusive [29]. *76. *Tt*LPMO9E: Inactive towards XG using ascorbic acid, but XG-active when using photosynthetic pigments with light [33]. *84. *Pa*LPMO9E: XG catalytic performance was determined based on a colorimetric H_2_O_2_-production assay [31]

To test our hypothesis whether the mode-of-action of AA9 LPMOs towards XG is dependent on the type of active site segments, as showcased by *Nc*LPMO9C and *Nc*LPMO9M (Fig. 8), amino acid sequence alignment and phylogenetic analysis were conducted. Here, all characterized fungal AA9 LPMOs (cellulose-active and XG-(plus cellulose)active LPMOs) and a number of randomly selected uncharacterized AA9 LPMOs from the CAZy database were compared. We first aligned the mature amino acid sequences (Additional file 2), which revealed three main clusters, and generated an unrooted “full-length” (FL) phylogenetic tree (Additional file 1: Fig. S7). The clustering of AA9 LPMOs into three groups has already been described in literature [36, 40, 41, 52–54], however, never been used for comparisons of active site segments and XG catalytic behavior. Next, only the amino acids of the five active site segments (Seg1–Seg5, based on the definition described in our previous study [41]) were aligned (Additional file 3) and subjected to a phylogenetic analysis. The resulting structure-based “segments-only” (SO phylogenetic tree (Additional file 1: Fig. S8; Fig. 9) shows three main clusters: one with the structural features ^+^Seg1^−^Seg2 (red area), the second defined as ^−^Seg1^+^Seg2 (light blue area) and the third defined as ^−^Seg1^−^Seg2 (yellow area). A sub-cluster with a ^−^Seg1^+^Seg2 feature was found (dark blue area in Fig. 9), but mostly with an extended Seg3 (^−^Seg1^+^Seg2^+^Seg3).

## Discussion

### Homology of active site segments of XG-active and XG-inactive LPMOs

As previously described, *Nc*LPMO9C and *Nc*LPMO9M have a different catalytic site configuration in terms of neighboring segments, in particular for Seg1 and Seg2 (Fig. 1). In this research, we characterized *Nc*LPMO9C as “Substitution-intolerant” and *Nc*LPMO9M as “Substitution-tolerant”. From this, we hypothesized that the long/short Seg1 and Seg2 is a generic feature amongst AA9 LPMOs altering their interaction with XG, which further steers their mode-of-action in degrading XG. Indeed, the characterized *Nc*LPMOs belong to different clusters of the structure-based SO phylogenetic tree of AA9 LPMOs (Fig. 9). Whether other characterized AA9 LPMOs, shown in the three clusters, have been reported to represent “Substitution-intolerant” or “Substitution-tolerant” oxidative cleavage activities is discussed here. Note that all discussed AA9 LPMOs are able to oxidatively cleave cellulose. For ease of structural comparison, published three-dimensional structures or homology models of selected characterized AA9 LPMOs from each of the three main phylogenetic clusters are shown in Additional file 1: Fig. S9.

Based on published HPAEC and MS data, multiple LPMOs clustering in the ^−^Seg1^+^Seg2 area (light blue area in Fig. 9) were reported to show “Substitution-intolerant” oxidative cleavage activities, like *Nc*LPMO9C [(blue dots, No. 43 in Fig. 9; Additional file 1: Fig. S9a), e.g., *Mt*LPMO9J (No. 41 in Fig. 9) [22], *Nc*LPMO9D (No. 51 in Fig. 9; Additional file 1: Fig. S9b) [20], *Cv*AA9A (No. 54 in Fig. 9; Additional file 1: Fig. S9c) and *Ls*AA9A (No. 56 in Fig. 9; Additional file 1: Fig. S9d) [24]. Again, mainly based on published HPAEC and MS data, LPMOs clustering in the ^+^Seg1^–^Seg2 area (red area in Fig. 9) were reported to show seemingly “Substitution-intolerant” behavior (red dots), as observed for *Gt*LPMO9A-2 (No. 7 in Fig. 9; Additional file 1: Fig. S9f) [28], *Fg*LPMO9A (No. 15 in Fig. 9; Additional file 1: Fig. S9g) [27], *Ta*LPMO9A (No. 31 in Fig. 9; Additional file 1: Fig. S9h) [21], *Gc*LPMO9B (No. 38 in Fig. 9) and *Gc*LPMO9A (No. 39 in Fig. 9) [25]. The latter enzymes all have an active site segment configuration comparable to *Nc*LPMO9M (Additional file 1: Fig. S9e).

It should be stressed that several other LPMOs have been reported to be active on XG [26, 29–31]; however, the corresponding published product profiles were not conclusive due to poor chromatographic or mass spectrometric representations (Fig. 9, green dots). In addition, several AA9 LPMOs have been shown to be inactive on XG alone (in brief “Inactive”; see references [20, 33] and Additional file 1: Fig. S10). Generally, we found that the ^−^Seg1^−^Seg2 configuration (Fig. 9, yellow area) promotes “Inactive” (e.g., *Nc*LPMO9F, No. 78 in Fig. 9; Additional file 1: Figs. S9i, S10).

Interestingly, we found that LPMOs having the structural feature of ^−^Seg1^+^Seg2^+^Seg3 (dark blue area in Fig. 9) also showed no activity on XG alone, e.g., *Mt*LPMO9B (No. 63 in Fig. 9; Additional file 1: Fig. S9j) [30] and *Mt*LPMO9I (No. 66 in Fig. 9; Additional file 1: Fig. S11). In the ^−^Seg1^+^Seg2^+^Seg3 cluster, although sharing the similar structural ^−^Seg1^+^Seg2 feature, the Seg3 segment is more extended (Additional file 3) compared to other LPMOs in the ^−^Seg1^+^Seg2 cluster [e.g., *Nc*LPMO9C (No. 43 in Fig. 9; Additional file 1: Fig. S9a), *Nc*LPMO9D (No. 51 in Fig. 9; Additional file 1: Fig. S9b), *Cv*AA9A (No. 54 in Fig. 9; Additional file 1: Fig. S9c) and *Ls*AA9A (No. 56 in Fig. 9; Additional file 1: Fig. S9d)]. In addition, our previous study demonstrated that LPMOs in the dark blue sub-cluster possess a cysteine in Seg2, which forms a disulfide bridge to a second cysteine in Seg3 [41]. This disulfide bridge may pull the Seg2 away from the active site, which could mimic the effect of a short Seg2 [41]. This “shortening” effect might also affect the catalytic performance towards XG as Seg2 cannot properly align to the XG alone. This could result in the described XG inactivity of LPMOs in this sub-cluster.

### Challenges in linking the mode-of-action of AA9 LPMOs to their active site segments

The AA9 structure-based phylogenetic tree (Fig. 9) showed three main clusters: (i) LPMOs with a ^−^Seg1^+^Seg2 configuration and following a “Substitution-intolerant” mode-of-action; (ii) LPMOs with a ^+^Seg1^−^Seg2 configuration and following a “Substitution-tolerant” one and (iii) LPMOs with a ^−^Seg1^+^Seg2 and a ^−^Seg1^+^Seg2^+^Seg3 configuration and showing only activity towards cellulose and no activity towards XG alone (Fig. 9). These correlations between protein structure and XG activity further reflect that AA9 LPMOs may require at least one long Seg1 or Seg2 to enable oxidative XG cleavage. Still, in each (sub-)cluster (Fig. 9) of the SO phylogenetic tree, beyond the challenge that a large number of reported LPMOs have not yet been tested for XG activity (Fig. 9, grey dots; [34, 37, 40, 52, 55–62]), irregularities seem to occur.

In the ^+^Seg1^−^Seg2 cluster (red area in Fig. 9), a “Substitution-tolerant” mode-of-action was found for all characterized LPMOs, except for *Pa*LPMO9D (No. 17 in Fig. 9), which was determined to be “Inactive”, although only based on a colorimetric H_2_O_2_-production assay [31]. A similar conclusion of “Inactive” for *Pa*LPMO9B (No. 62 in Fig. 9) and *Pa*LPMO9E (No. 84 in Fig. 9) was drawn also based on the H_2_O_2_-production assay [31]. As only a repression of the H_2_O_2_ production of the LPMOs is measured with this peroxidase assay, it cannot be concluded whether these LPMOs show really no oxidative cleavage of XG. Hence, to confirm their (non-) XG activity, a more detailed chromatography- and mass spectrometry-based analysis is required.

In the cluster of ^−^Seg1^+^Seg2 (light blue area in Fig. 9), *Nc*LPMO9A (No. 47 in Fig. 9), having a high structural similarity to *Nc*LPMO9C (No. 43 in Fig. 9) and *Nc*LPMO9D (No. 51 in Fig. 9), displayed no activity on XG alone [20]. *Nc*LPMO9A showed the “Substitution-intolerant” degradation only when cellulose was present [20] and apparently is, an exception in this cluster. From the same cluster, *Pa*LPMO9H (No. 45 in Fig. 9) was reported as “Substitution-tolerant” LPMO by using direct infusion mass spectrometry [26]. But, in another research, the HPAEC chromatogram of a *Pa*LPMO9H-TXG-digest showed a more “Substitution-intolerant” behavior [31]. Again, a more detailed chromatography- and mass spectrometry-based analysis is required to unambiguously define the mode-of-action of *Pa*LPMO9H towards XG. Nevertheless, taking a closer look at the *Pa*LPMO9H structure, it appeared that this enzyme has a higher content of hydrophobic amino acid residues (F, W, Y) in Seg1, less charged residues but a higher negative net charge in Seg3, and one additional positively charged residue in Seg4 (Additional files 2 and 3), compared to *Nc*LPMO9C.

Also, in the ^−^Seg1-^+^Seg2 cluster (yellow area in Fig. 9) some exceptions were annotated. For example, AN3046 (No. 73 in Fig. 9) was reported to be active towards XG based on MALDI-TOF-MS data [29]. However, these data remain to be verified with other analytical techniques, as the reported MALDI-TOF mass spectra only showed aldonic acid forms, while *m/z*-values of δ-lactone forms were absent. Detection of aldonic acids without δ-lactones in MALDI-TOF-MS analysis of LPMO-digests has not been observed in other studies. In addition, only XXLG^ox^ and XLLG^ox^ were detected in the LPMO-TXG digest, while the more common XXXG^ox^ block was not found [29]. Another still difficult to classify candidate in the ^−^Seg1^−^Seg2 cluster (yellow area in Fig. 9) is *Tt*LPMO9E (No. 76 in Fig. 9), which has been reported as “Inactive” when using Asc as electron donor, but as active when reduced by photosynthetic pigments with light [33]. The above special cases, together with LPMOs not yet tested on XG, further exemplify the difficulties and pitfalls in understanding LPMO mode-of-action towards XG based on their active site segment configuration. The latter can only be properly understood if not only experimental conditions and assays used are carefully considered, but also detailed characterization of LPMO-XG degradation products is performed, which further reflects the importance of our research. Hence, careful characterization of more LPMO mode-of-actions towards XG is highly recommended to further understand how active site segments steer the XG degradation by AA9 LPMOs.

## Conclusions

In this study, we described two distinct XG degradation patterns generated by two AA9 *Nc*LPMOs representing different configuration of active site segments. The oxidative cleavage of XG by *Nc*LPMO9C predominantly occurred at the non-reducing end of single unbranched glucosyl units (“Substitution-intolerant”), while *Nc*LPMO9M displayed a more substitution-tolerant cleavage behavior (“Substitution-tolerant”). Based on active site segment phylogeny of AA9 LPMOs, “Substitution-intolerant” was found to correlate to the configuration ^−^Seg1^+^Seg2, while “Substitution-tolerant” correlated to ^+^Seg1^−^Seg2. These findings support the hypothesis that the mode-of-action of AA9 LPMOs towards XG is based on the distinct structural features of their active site segments.

## Materials and methods

### XG substrates, carbohydrate standards and other chemicals

XG from tamarind (*Tamarindus indica*, TXG) seed, TXG oligosaccharide standards (xyloglucan hepta + octa + nona saccharides) and XEG (GH5) from *Paenibacillus* sp. were purchased from Megazyme (Bray, Ireland). XG from black currants (*Ribes nigrum L.*, BCXG) was available in our laboratory (fraction CASS) extracted by Hilz and coworkers [17]. Glucose was purchased from Sigma-Aldrich (St. Louis, Missouri, USA) and Asc was purchased from VWR International (Radnor, PA, USA). Cellobiose, cellotriose, cellotetraose, cellopentaose and cellohexaose were used as standards and purchased from Megazyme. Water used in all experiments was generated by a Milli-Q system (Millipore, Molsheim, France), unless mentioned otherwise.

### Catalytic performance of XEG, *Nc*LPMO9C and *Nc*LPMO9M on XG

Expression, production and purification of *Nc*LPMO9C and *Nc*LPMO9M were described previously [41]. XG substrates (TXG or BCXG, 2 mg/mL) were dissolved in 50 mM ammonium acetate buffer (pH 5.0) with the addition of Asc (1 mM final concentration). Subsequently, XEG, *Nc*LPMO9C and *Nc*LPMO9M were added to a concentration of 1.25 µM. Control reactions were performed without the addition of Asc. Single 200 µL reactions were incubated in an Eppendorf ThermoMixer^®^ C at 800 rpm (in a vertical orientation) and reactions used to produce the time curves were incubated in a head-over-tail rotator at 20 rpm (5 mL total volume). *Nc*LPMO9C and *Nc*LPMO9M reactions were incubated at 30 °C while XEG reaction was at 50 °C. All reactions were performed in duplicate. To create a time curve for *Nc*LPMO9C and *Nc*LPMO9M, a larger reaction volume of 500 µL was sampled at 0, 1, 2, 4, 8 and 24 h after enzyme addition. The reactions were stopped while incubating for 10 min at 97 °C in an Eppendorf ThermoMixer^®^ C. Subsequently, the supernatant was recovered after centrifugation in a Hermile Z 233 MK-2 centrifuge at 22000×*g* (Rotor: 220.87 VO5/6) for 20 min and stored at − 20 °C until further usage. Parts of XEG- and *Nc*LPMO9C-TXG-digests were further treated with β-galactosidase (GH35 from *Aspergillus niger*, Megazyme), which is further described in Additional file 1.

### Analytical methods

#### HPSEC analysis for molecular weight distribution of (degraded) TXG

TXG and corresponding digests were analyzed by HPSEC-RI for their molecular weight distribution. Instrument settings, column and elution program were the same as described previously [41]. Pullulans (Associated Polymer Labs Inc., New York, USA) in the MW range of 0.4–708 kDa were used for calibration.

#### HPAEC analysis for profiling oligosaccharides

TXG and corresponding digests were analyzed by HPAEC-PAD on an ICS5000 (Dionex) system equipped with a CarboPac PA-1 column (2 mm ID × 250 mm) in combination with a CarboPac PA guard column (2 mm ID × 50 mm). Mobile phases were (A) 0.1 M NaOH and (B) 1 M NaOAc in 0.1 M NaOH. The column temperature was 20 °C. The elution program applied has been described previously [30]. Samples were diluted five times before analysis. Commercial TXG oligosaccharide mixture (50 µg/mL), glucose (2.5 µg/mL) and cellodextrins (DP 2–6, 2.5 µg/mL) were used as standards.

#### MALDI-TOF-MS analysis of oligosaccharides

To analyze the mass of formed XG oligosaccharides, MALDI-TOF-MS (Bruker Daltonics, Billerica, Massachusetts, USA) was used as previously described [47]. The mass spectrometer was calibrated using maltodextrins (Avebe, Veendam, The Netherlands) in a mass range (*m/z*) of 500–3000 and a total of 300 spectra were collected for each measurement. Prior to analysis, samples were desalted using Dowex AG 50 W-X8 Resin (Bio-Rad Laboratories, Hempel Hempstead, UK). The desalted supernatants were dried under nitrogen and re-dissolved in water containing 20 mM LiCl to obtain lithium (Li)-adducts. 1 µL of each lithium-rich sample was mixed with 1 µL matrix solution (50% (v/v) acetonitrile in H_2_O containing 12 mg/mL 2,5-dihydroxy-benzoic acid (Bruker Daltonics)) and dried under nitrogen.

#### HILIC-ESI-CID-MS/MS for structural elucidation of (degraded) XG

The LPMO-TXG- and -BCXG-digests were separated and analyzed using HILIC coupled to ESI-MS. To separate the TXG oligosaccharides, a Vanquish UHPLC system (Thermo Scientific, San Jose, CA, USA) equipped with an Acquity UPLC BEH Amide column (1.7 μm, 2.1 mm ID × 150 mm) and a VanGuard pre-column (1.7 μm, 2.1 mm ID × 5 mm) was used. Supernatants from LPMO-TXG- and LPMO-BCXG-digests were concentrated five times and then subjected (2 μL) to the column. The column temperature was set at 35 °C using the still air mode and the flow rate was 0.45 mL/min. Water (A) and acetonitrile (B) both containing 0.1% formic acid (all were UHPLC-grade; Biosolve, Valkenswaard, The Netherlands) were used as mobile phases. The elution profile was: 0–2 min at 82% B (isocratic), 2–62 min from 82% to 60% B (linear gradient), 62–62.5 min from 60% to 42% B (linear gradient), 62.5–69 min at 42% B (isocratic), 69–70 min from 42% to 82% B (linear gradient) and 70–80 min at 82% B (isocratic). The MS settings have been described previously [51]. The full MS (*m/z*) range was set to 300–2000. To improve the fragmentation, MS/MS was performed using dependent scan followed by a parent mass list. The mass list used is displayed in Additional file 1: Table S1. For MS/MS, the CID with a normalized collision energy was set at 35%, the minimum signal threshold was 20,000 counts, activation Q was 0.15 and activation time was 10 ms. Mass spectrometric data were processed using Xcalibur 2.2 (Thermo Scientific).

### Crystal structures and homology models

Structural data of LPMOs were derived from the RCSB protein data bank (https://www.rcsb.org). Homology models of LPMOs without published three-dimensional structures were generated using SWISS-MODEL (https://swissmodel.expasy.org) [63–67]. Template search with BLAST [68] and HHBlites [69] were performed against the SWISS-MODEL template library (SMTL). The target sequences were searched with BLAST against the primary amino acid sequence contained in the SMTL. The PyMOL Molecular Graphics System (Version 1.7.2.1 Schrödinger, LLC) was used for visualization and structural alignments.

### Sequence mining, structure-based multiple sequence alignment and phylogenetic analysis

In order to obtain an unbiased set of amino acid sequences, which covers the whole range of the large variety within AA9 LPMOs, sequences were selected randomly from the 498 available eukaryotic AA9 LPMO sequences in the CAZy database. This set was completed by addition of all AA9 LPMO sequences labeled as “characterized” in the CAZy database, all AA9 LPMO sequences with a resolved structure, and those with known XG (in)activity, if not already present in the set. The amino acid sequences were aligned using the MUSCLE algorithm [70] in MEGA7 [71] and fine-tuned by cutting out the signal peptide, the linker- and the CBM-region, as well as sequences not fitting to the alignment. The amino acid sequences were then realigned using the structure-based MAFFT-DASH algorithm [72]. The resulting structure-based alignment was then cut down to the regions of interest termed “Segments 1 to 5” (Seg1–Seg5).

Phylogenetic analysis of both the FL and SO structure-based multiple sequence alignment was done using RAxML-NG [73]. Firstly, the alignments were tested for the most applicable substitution model using ModelTest-NG [74]. The tree was inferred using the BLOSUM62 model [75] (number of discrete gamma categories: 4; with frequencies and invariant sites) for the FL alignment, and the Probability Matrix from Blocks (PMB) [76] model (number of discrete gamma categories: 4; with frequencies and invariant sites) for the SO alignment and 20 starting trees were calculated. Bootstrap analysis was then carried out until convergence criteria (cut-off: 0.03) based on the bootstopping test [77] were reached (800 and 1120 bootstraps for the FL and SO alignment, respectively). The resulting phylogenetic trees were prepared for publication using MEGA7.

## Supplementary information

**Additional file 1. Fig. S1.** MW distributions of TXG-digests from *Nc*LPMO9C and *Nc*LPMO9M. **Fig. S2.** HPAEC elution patterns of β-galactosidase treated XEG and *Nc*LPMO9C digested TXG. **Fig. S3.** MALDI-TOF mass spectra of β-galactosidase treated XEG and *Nc*LPMO9C digested TXG. **Fig. S4.** Negative ion mode CID-MS/MS fragmentation patterns of XXX and XLL present in the *Nc*LPMO9C-TXG-digest. **Fig. S5.** Negative ion mode CID-MS/MS fragmentation patterns of XGX and LXG present in the *Nc*LPMO9M-TXG-digest. **Fig. S6.** HILIC-ESI-MS base-peak chromatograms of BCXG-digests from *Nc*LPMO9C and *Nc*LPMO9M. **Fig. S7.** Phylogenetic tree in circle style of “full length” (FL) AA9 LPMOs. **Fig. S8.** Phylogenetic tree in circle style of “segments only” (SO) AA9 LPMOs. **Fig. S9.** Crystal structures and homology models of LPMOs tested on XG. **Fig. S10.** HPAEC elution patterns of TXG, and of TXG digested with *Nc*LPMO9F with the addition of Asc. **Fig. S11.** HPAEC elution patterns of TXG, and of TXG digested with *Mt*LPMO9I with the addition of Asc. **Table S1.** Selected mass (*m/z*) list for non-, C4- and C1-oxidized TXG oligosaccharides. **Table S2.** List of non-oxidized XG oligosaccharides identified based on fragmentation patterns in CID-MS/MS present in the *Nc*LPMO9C-TXG-digest. **Table S3.** List of non-oxidized XG oligosaccharides identified based on fragmentation patterns in CID-MS/MS present in the *Nc*LPMO9M-TXG-digest. **Table S4.** Characterized LPMOs; organism, Genbank accession number, LPMO name (if applicable), PDB entry (if applicable) and reference (if applicable).

**Additional file 2.** Structure-based amino acid “full sequence” alignment of AA9 LPMOs.

**Additional file 3.** Structure-based amino acid “segments only” sequence alignment of AA9 LPMOs.

## Data Availability

All data generated or analyzed during this study are included in this published article and its additional files.

## Abbreviations

XG: Xyloglucan
*Nc*: *Neurospora crassa*
LPMOs: Lytic polysaccharide monooxygenases
TXG: Tamarind seed xyloglucan
BCXG: Black currant xyloglucan
CAZy: Carbohydrate-active enzymes database
AA9: Auxiliary activities family 9
HPAEC-PAD: High-performance anion exchange chromatography-pulsed amperometric detection
XEG: Xyloglucanase (GH5)
MALDI-TOF-MS: Matrix assisted laser desorption/ionization-time of flight-mass spectrometry
H_n_P_m_: “H” for “hexaose”, “P” for “pentaose”, “n” for the number of hexaoses and “m” for the number of pentaoses
HILIC-ESI-CID-MS/MS: Hydrophilic interaction chromatography-electrospray ionization-collision induced dissociation-mass spectrometry
MW: Molecular weight
HPSEC-RI: High-performance size exclusion chromatography-refractive index detection
Asc: Ascorbic acid
DP: Degree of polymerization
FL: Full sequence
SO: Segments only
RAxML-NG: Randomized axelerated maximum likelihood-next generation
PMB: Probability matrix from blocks
