# Supplementary material for: Configuration of active site segments in lytic polysaccharide monooxygenases steers oxidative xyloglucan degradation
Source: Biotechnol Biofuels. 2020 May 29;13:95. doi: 10.1186/s13068-020-01731-x (PMC7257166; doi:10.1186/s13068-020-01731-x)
Supplement: Supplementary file 1 — Additional file 1. Fig. S1. MW distributions of TXG-digests from NcLPMO9C and NcLPMO9M. Fig. S2. HPAEC elution patterns of β-galactosidase treated XEG and NcLPMO9C digested TXG. Fig. S3. MALDI-TOF mass spectra of β-galactosidase treated XEG and NcLPMO9C digested TXG. Fig. S4. Negative ion mode CID-MS/MS fragmentation patterns of XXX and XLL present in the NcLPMO9C-TXG-digest. Fig. S5. Negative ion mode CID-MS/MS fragmentation patterns of XGX and LXG present in the NcLPMO9M-TXG-digest. Fig. S6. HILIC-ESI-MS base-peak chromatograms of BCXG-digests from NcLPMO9C and NcLPMO9M. Fig. S7. Phylogenetic tree in circle style of “full length” (FL) AA9 LPMOs. Fig. S8. Phylogenetic tree in circle style of “segments only” (SO) AA9 LPMOs. Fig. S9. Crystal structures and homology models of LPMOs tested on XG. Fig. S10. HPAEC elution patterns of TXG, and of TXG digested with NcLPMO9F with the addition of Asc. Fig. S11. HPAEC elution patterns of TXG, and of TXG digested with MtLPMO9I with the addition of Asc. Table S1. Selected mass (m/z) list for non-, C4- and C1-oxidized TXG oligosaccharides. Table S2. List of non-oxidized XG oligosaccharides identified based on fragmentation patterns in CID-MS/MS present in the NcLPMO9C-TXG-digest. Table S3. List of non-oxidized XG oligosaccharides identified based on fragmentation patterns in CID-MS/MS present in the NcLPMO9M-TXG-digest. Table S4. Characterized LPMOs; organism, Genbank accession number, LPMO name (if applicable), PDB entry (if applicable) and reference (if applicable). [file 13068_2020_1731_MOESM1_ESM.docx]

**Additional Information 1**

**Configuration of active site segments in lytic polysaccharide monooxygenases steers oxidative xyloglucan degradation**

**Authors**

*****Peicheng Sun^a^, *****Christophe V.F.P. Laurent^b,c^, Stefan Scheiblbrandner^b^, Matthias Frommhagen^a^, Dimitrios Kouzounis^a^, Mark G. Sanders^a^, Willem J.H. van Berkel^a^, Roland Ludwig^b^, Mirjam A. Kabel^a, #^

**Affiliations**

^a^Laboratory of Food Chemistry, Wageningen University & Research, Bornse Weilanden 9, 6708 WG Wageningen, The Netherlands.

^b^Biocatalysis and Biosensing Laboratory, Department of Food Science and Technology, BOKU-University of Natural Resources and Life Sciences, Vienna, Muthgasse 18, 1190 Vienna, Austria

^c^Institute of Molecular Modelling and Simulation, Department of Material Sciences and Process Engineering, BOKU-University of Natural Resources and Life Sciences, Vienna, Muthgasse 18, 1190 Vienna, Austria

***These authors contributed equally to this work.**

^#^Corresponding author (Telephone: +31 (0)317 48 32 09, email: mirjam.kabel@wur.nl)

**Additional Information 1 content:**

**Additional materials and methods**

**Fig. S1.** MW distributions of TXG-digests from *Nc*LPMO9C and *Nc*LPMO9M

**Fig. S2.** HPAEC elution patterns of β-galactosidase treated XEG and *Nc*LPMO9C digested TXG

**Fig. S3.** MALDI-TOF mass spectra of β-galactosidase treated XEG and *Nc*LPMO9C digested TXG

**Fig. S4.** Negative ion mode CID-MS/MS fragmentation patterns of XXX and XLL present in the *Nc*LPMO9C-TXG-digest

**Fig. S5.** Negative ion mode CID-MS/MS fragmentation patterns of XGX and LXG present in the *Nc*LPMO9M-TXG-digest

**Fig. S6.** HILIC-ESI-MS base-peak chromatograms of BCXG-digests from *Nc*LPMO9C and *Nc*LPMO9M

**Fig. S7.** Phylogenetic tree in circle style of “full length” (FL) AA9 LPMOs

**Fig. S8.** Phylogenetic tree in circle style of “segments only” (SO) AA9 LPMOs

**Fig. S9.** Crystal structures and homology models of LPMOs tested on XG

**Fig. S10.** HPAEC elution patterns of TXG, and of TXG digested with *Nc*LPMO9F with the addition of Asc

**Fig. S11.** HPAEC elution patterns of TXG, and of TXG digested with *Mt*LPMO9I with the addition of Asc

**Table S1.** Selected mass (m/z) list for non-, C4- and C1-oxidized TXG oligosaccharides

**Table S2.** List of non-oxidized XG oligosaccharides identified based on fragmentation patterns in CID-MS/MS present in the NcLPMO9C-TXG-digest

**Table S3.** List of non-oxidized XG oligosaccharides identified based on fragmentation patterns in CID-MS/MS present in the NcLPMO9M-TXG-digest

**Table S4.** Characterized LPMOs; organism, Genbank accession number, LPMO name (if applicable), PDB entry (if applicable) and reference (if applicable)

**References**

**Additional materials and methods**

**Materials**

Isoprimeverose (X unit) and β-galactosidase (GH35) from *Aspergillus niger* were purchased from Megazyme (Bray, Ireland). Other carbohydrate substrates, standards and chemicals were described in the section “Materials and methods”.

**Removal of galactosyl residues in TXG oligosaccharides by β-galactosidase**

Stock β-galactosidase (4000 U/mL) was diluted with 100 mM sodium acetate buffer (pH 4.0) to 4 U/mL. Afterwards, 60 µL of diluted β-galactosidase solution was added to 180 µL of XEG- and *Nc*LPMO9C-TXG-digests (prepared as described in the section 5.2), respectively. The reactions were incubated at 30 °C for 24 h under shaking at 800 rpm in an Eppendorf ThermoMixer® C. After 24 h incubation, the reactions were stopped at 97 °C for 10 min in an Eppendorf ThermoMixer® C. Subsequently, the supernatant was recovered after centrifugation in a Hermile Z 233 MK-2 centrifuge at 22000 × *g* (Rotor: 220.87 VO5/6) for 10 min. The β-galactosidase treated XEG- and *Nc*LPMO9C-TXG-digests were analyzed by HPAEC-PAD and MALDI-TOF-MS with the same settings described in the section “Materials and methods”. TXG oligosaccharide standards (100 µg/mL) and isoprimeverose (X unit, 50 µg/mL) were included in the analysis as standards.

**
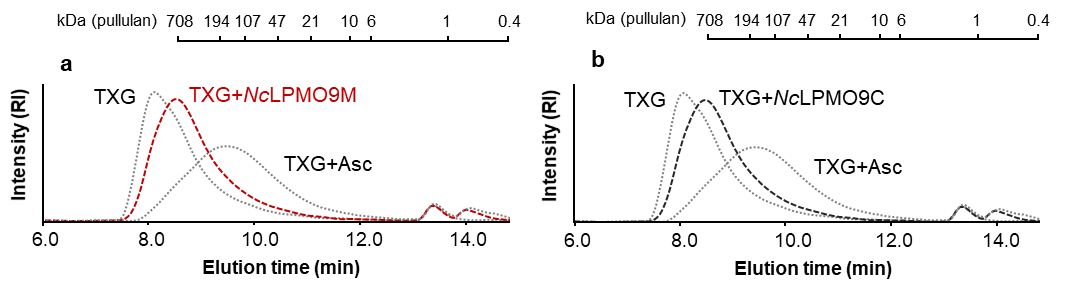
Fig. S****1**. Elution patterns that show the molecular weight (MW) distributions of tamarind seed xyloglucan (TXG) digests from (a) *Nc*LPMO9M (1.25 μM, TXG + *Nc*LPMO9M) and (b) *Nc*LPMO9C (1.25 μM, TXG + *Nc*LPMO9C) in the absence of ascorbic acid (Asc), analyzed by HPSEC-RI. For comparison, TXG without enzyme-addition, but with Asc (24 h; TXG + Asc) is also shown.

**
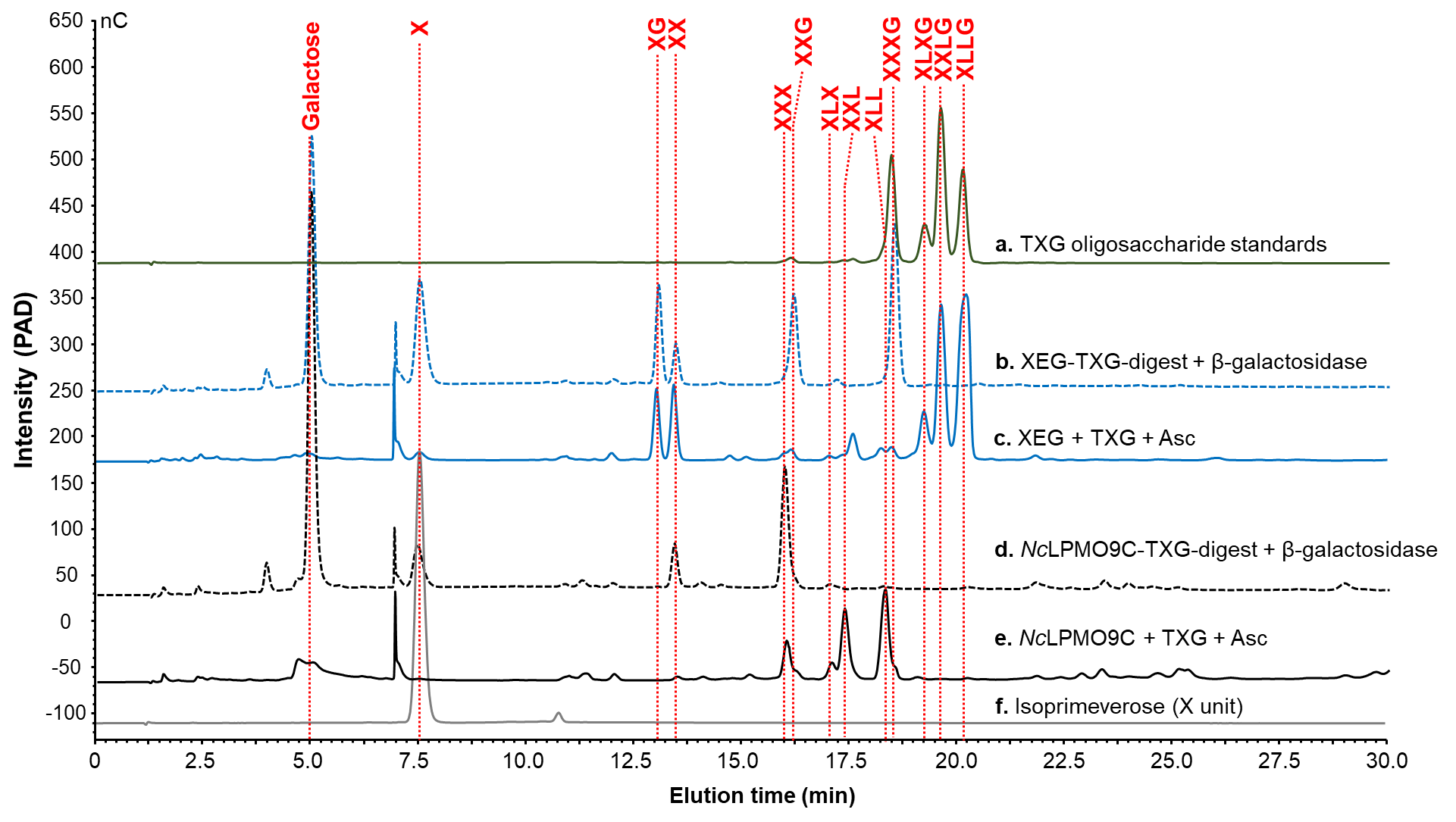
Fig. S2**. HPAEC elution patterns of products after β-galactosidase treatment of xyloglucanase (XEG; c) and *Nc*LPMO9C (d) digested tamarind seed xyloglucan (TXG) in the presence of ascorbic acid (Asc). XEG- (b) and *Nc*LPMO9C-TXG-digests (d) before β-galactosidase treatment are included for comparison. Likewise, TXG oligosaccharide standards (a) and isoprimeverose (X unit; f) are also shown.

**
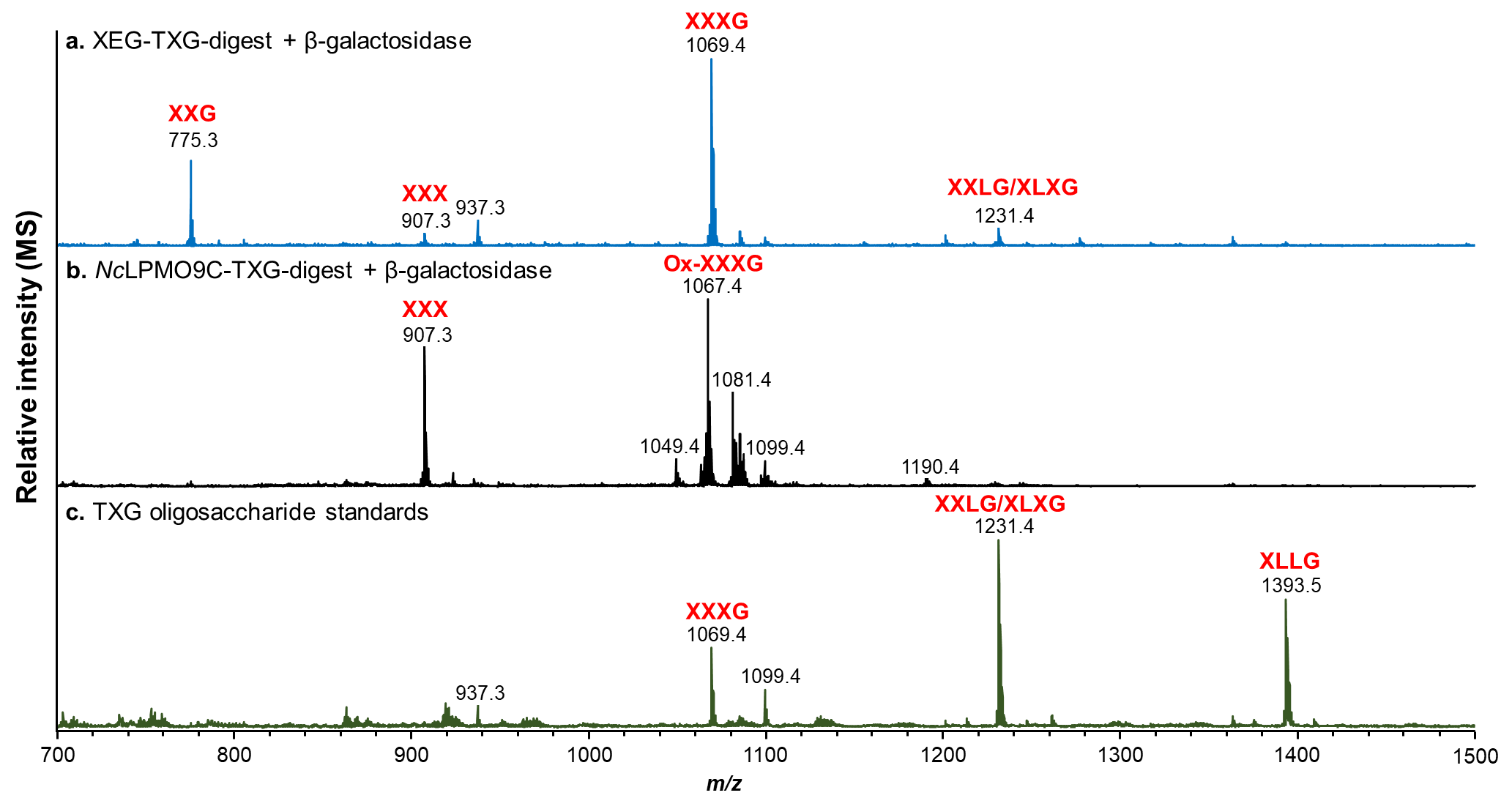
Fig. S3**. MALDI-TOF mass spectra of β-galactosidase-treated xyloglucanase (XEG; a) and *Nc*LPMO9C (b) digested tamarind seed xyloglucan (TXG) in the presence of ascorbic acid (Asc). The TXG oligosaccharide standards (c) are shown for comparison. Ox, oxidized. *M/z*-value corresponded to lithium (Li)-adducts.

**
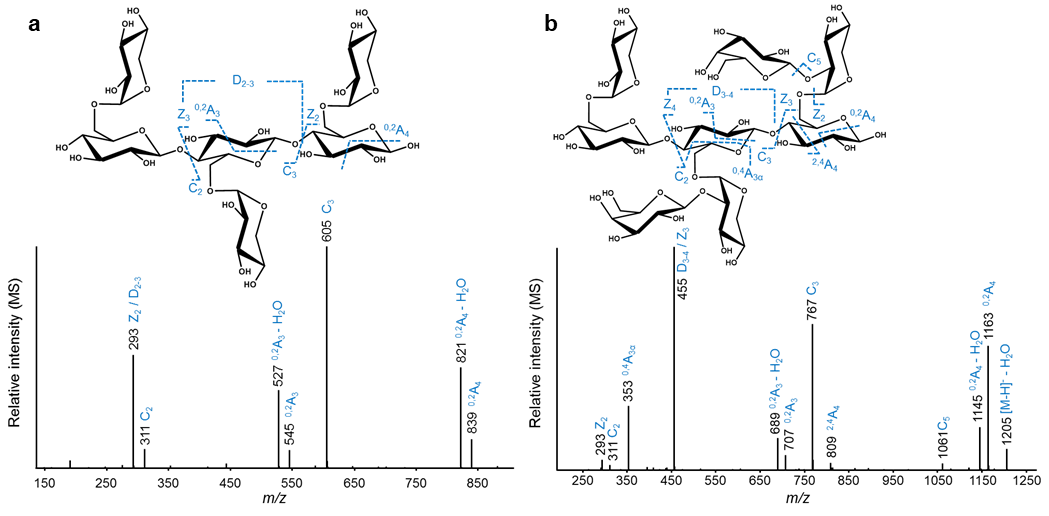
Fig. S4**. Negative ion mode CID-MS/MS fragmentation patterns of non-oxidized tamarind seed XG oligosaccharides present in the *Nc*LPMO9C-TXG-digest annotated as XXX (*m/z* 899.3, a) and XLL (*m/z* 1223.5, b). The fragments are annotated according to the nomenclature proposed by Domon & Costello (1).

**
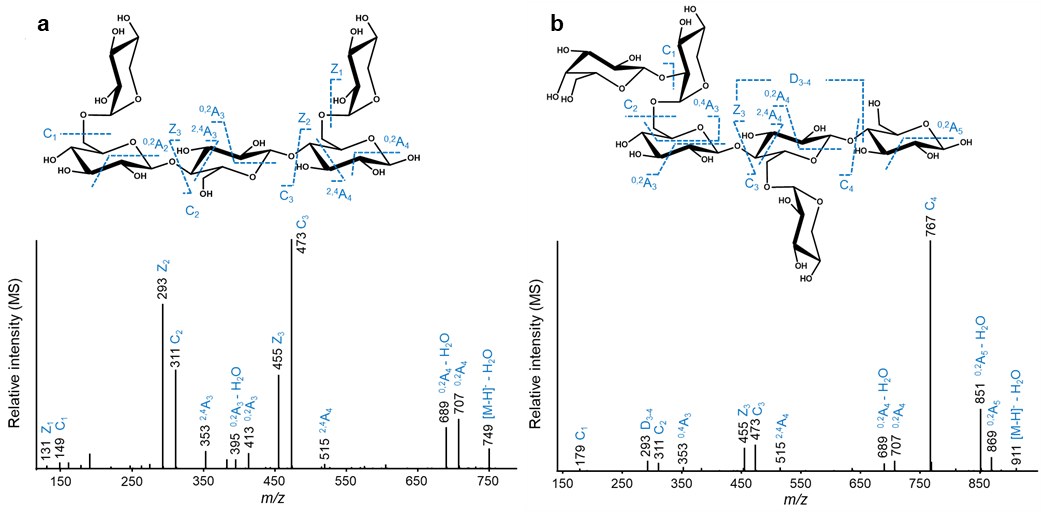
Fig. S5**. Negative ion mode CID-MS/MS fragmentation patterns of non-oxidized tamarind seed XG oligosaccharides present in the *Nc*LPMO9M-TXG-digest annotated as XGX (*m/z* 767.4, a) and LXG (*m/z* 929.3, b). The fragments are annotated according to the nomenclature proposed by Domon & Costello (1).

**
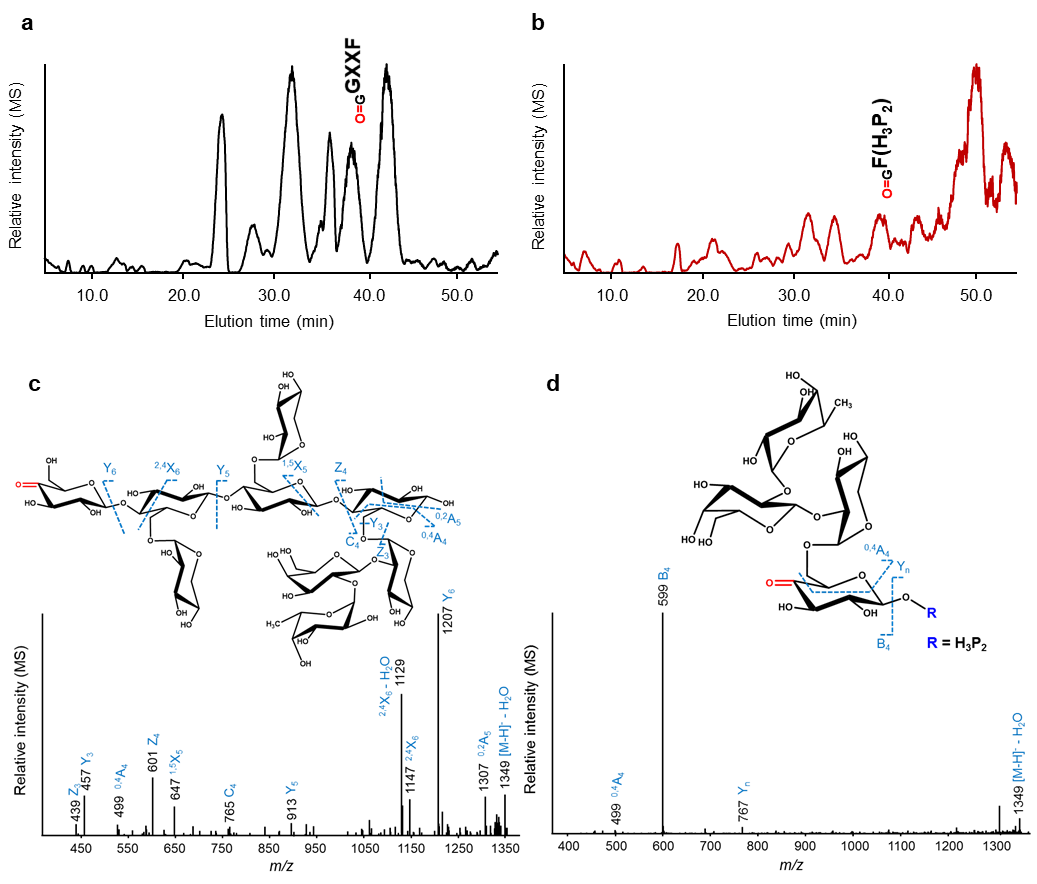
Fig. S6**. HILIC-ESI-MS base-peak chromatograms of black currant xyloglucan (BCXG) digested by (a) *Nc*LPMO9C and (b) *Nc*LPMO9M. Negative ion mode CID-MS/MS fragmentation patterns of C4-oxidized BCXG oligosaccharides present in *Nc*LPMO9C-BCXG-digest annotated as _O=G_GXXF (*m/z* 1367.7, c) and in *Nc*LPMO9M-BCXG-digest annotated as _O=G_F(H_3_P_2_) (*m/z* 1367.7, d). _O=G_ indicates that the oxidation is on the glucosyl unit in keto-form. Oxidation of the C4-carbon position is indicated in red. Products formed indicated that C4-oxidative cleavage occurred next to the F unit by *Nc*LPMO9M, but not by *Nc*LPMO9C. The fragments are annotated according to the nomenclature proposed by Domon & Costello (1).

**Fig. S7**. Phylogenetic tree (RaxML, 800 bootstraps) in circle style of “full length” (FL) (without signal peptide) AA9 LPMOs specified by their GenBank accession number. The Protein Data Bank (PDB) accession code as well as the possession of a carbohydrate binding module (+CBM) are indicated where it applies. Bootstrap values and evolutionary distance scale are indicated.

**Fig. S8**. Phylogenetic tree (RaxML, 1120 bootstraps) in circle style of “segments only” (SO) AA9 LPMOs specified by their GenBank accession number. The Protein Data Bank (PDB) accession code as well as the possession of a carbohydrate binding module (+CBM) are indicated where it applies. Bootstrap values and evolutionary distance scale are indicated.

**
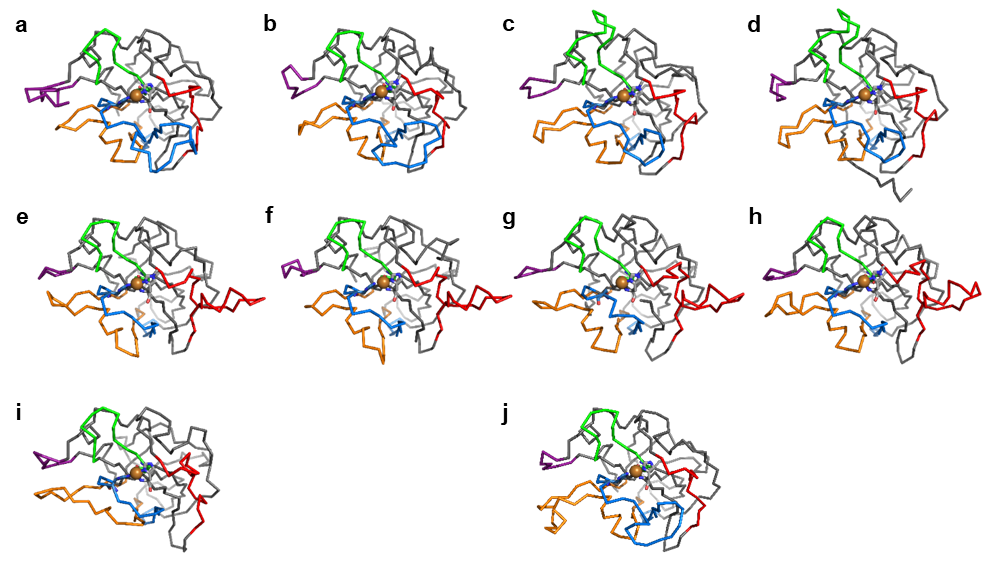
Fig. S9**. Crystal structures and homology models of LPMOs tested on xyloglucan. Seg1 to Seg5 are indicated in red, blue, yellow, green and purple, respectively. (a) *Nc*LPMO9C (EAA36362.1, “Substitution-intolerant”, PDB entry: 4D7U), (b) *Nc*LPMO9D (CAD21296.1, “Substitution-intolerant”, PDB entry: 4EIR), (c) *Cv*AA9A (AST24379.1, “Substitution-intolerant”, PDB entry: 5NLT), (d) *Ls*AA9A (ALN96977.1, “Substitution-intolerant”, PDB entry: 5ACF), (e) *Nc*LPMO9M (EAA33178.1, “Substitution-tolerant”, PDB entry: 4EIS), (f) *Gt*LPMO9A-2 (BAV57612.1, “Substitution-tolerant”, SwissModel SegID 59.09% with *Nc*LPMO9M), (g) *Fg*LPMO9A (CEF74660.1, “Substitution-tolerant”, SwissModel SegID 48.89% with *Tr*LPMO9A, CAA71999.1, PDB entry: 5O2W), (h) *Ta*LPMO9A (ACS5720.1, “Substitution-tolerant”, PDB entry: 2YET), (i) *Nc*LPMO9F (CAD70347.1, “Inactive”, PDB entry: 4QI8). (j) *Mt*LPMO9B (AON76800.1, “Inactive”, SegID 41.86% with *Nc*LPMO9D, EAA32426.1, PDB entry: 5TKF)


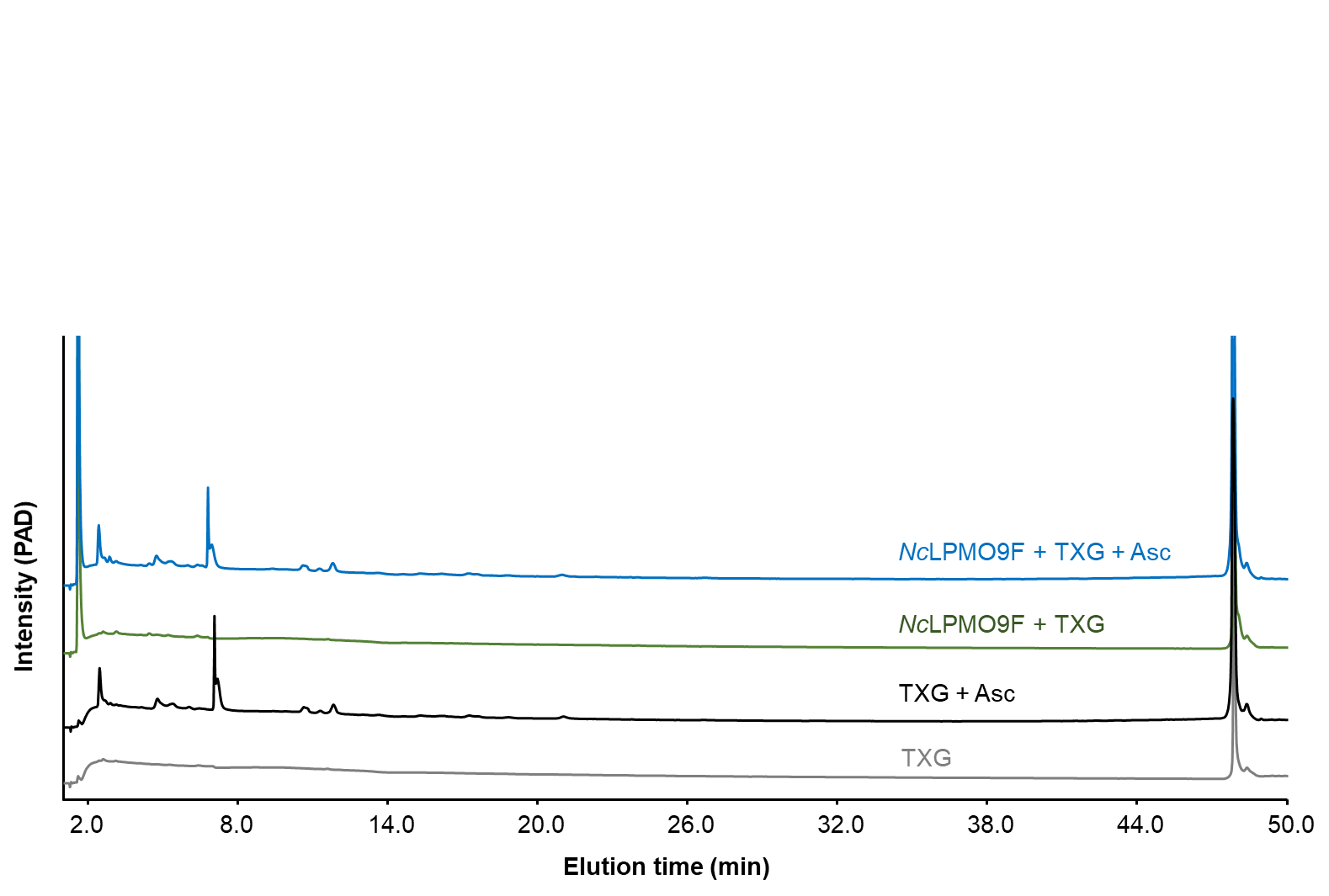


**Fig. S10**. HPAEC elution patterns of tamarind seed xyloglucan (TXG), and of TXG digested with *Nc*LPMO9F (1.25 μM) with addition of 1 mM ascorbic acid (Asc). Control reactions are TXG with *Nc*LPMO9F in the absence of Asc and TXG with only Asc. The chromatograms show no release of oxidized products by *Nc*LPMO9F. The expression, production and purification of *Nc*LPMO9F were described by Kittl et al. (2).

**
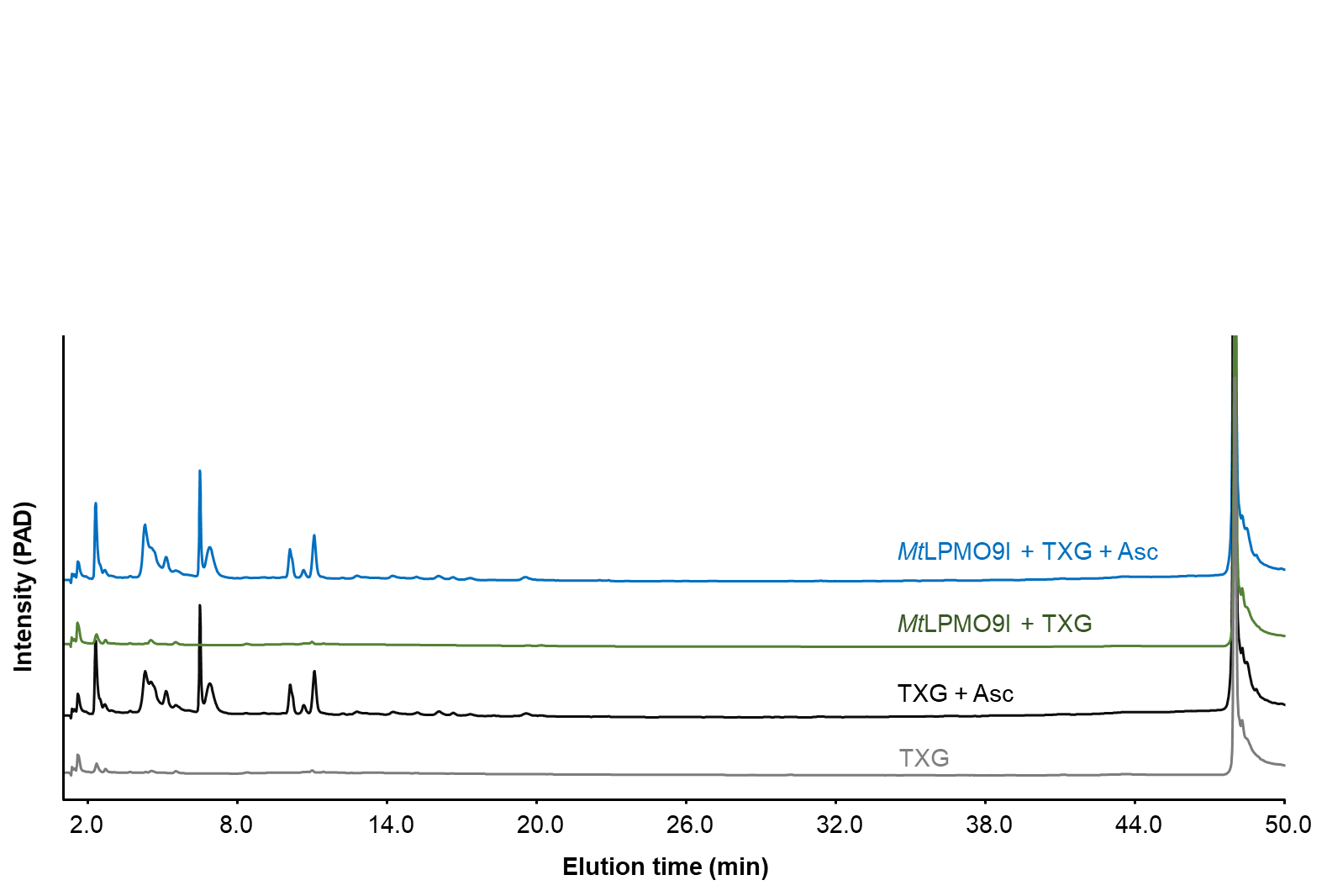
Fig. S11**. HPAEC elution patterns of tamarind seed xyloglucan (TXG), and of TXG digested with *Mt*LPMO9I (1.25 μM) with addition of 1 mM ascorbic acid (Asc). Control reactions are TXG with *Mt*LPMO9I in the absence of Asc and TXG with only Asc. The chromatograms show no release of oxidized products by *Mt*LPMO9I. The expression, production and purification of *Mt*LPMO9I were described by Sun et al. (3).

|  | **Non-Ox-TXG** | | **C4-Ox-TXG** | | **C1-Ox-TXG** | **Hex** | **Pen** |
| --- | --- | --- | --- | --- | --- | --- | --- |
|  | **[M - H]^-^** | **[M + FA-H]^-^** | **[M - 2 - H]^-^** | **[M - 2 + FA-H]^-^** | **[M + 16 - H]^-^** |  |  |
| *m/z* | 311.1 | 357.1 | 309.1 | 355.1 | 327.1 | 1 | 1 |
| *m/z* | 473.2 | 519.2 | 471.2 | 517.2 | 489.2 | 2 | 1 |
| *m/z* | 605.2 | *651.2 | 603.2 | 649.2 | 621.2 | 2 | 2 |
| *m/z* | 635.3 | 681.3 | 633.3 | 679.3 | *651.2 | 3 | 1 |
| *m/z* | 767.4 | 813.4 | 765.4 | 811.4 | 783.4 | 3 | 2 |
| *m/z* | 899.3 | *945.4 | 897.3 | 943.4 | 915.3 | 3 | 3 |
| *m/z* | 929.3 | 975.3 | 927.3 | 973.3 | *945.4 | 4 | 2 |
| *m/z* | 1061.4 | *1107.3 | 1059.4 | 1105.3 | 1077.4 | 4 | 3 |
| *m/z* | 1091.3 | 1137.3 | 1089.1 | 1135.3 | *1107.3 | 5 | 2 |
| *m/z* | 1223.5 | 1269.4 | 1221.5 | 1267.4 | 1239.5 | 5 | 3 |
| *m/z* | 1355.6 | *1401.6 | 1353.6 | 1399.6 | 1371.6 | 5 | 4 |
| *m/z* | 1385.7 | 1431.6 | 1383.7 | 1429.6 | *1401.6 | 6 | 3 |
| *m/z* | 1517.6 | 1563.6 | 1515.5 | 1561.4 | 1531.5 | 6 | 4 |
| *m/z* | 1679.6 | 1725.6 | 1677.6 | 1723.6 | 1695.6 | 7 | 4 |
| *m/z* | 1843.6 | 1889.6 | 1841.6 | 1887.6 | 1859.6 | 8 | 4 |

**Table S1**. Selected mass (*m/z*) list for non-oxidized (non-Ox), C4-oxidized (C4-Ox) and C1-oxidized (C1-Ox) TXG oligosaccharides (CID-MS/MS fragmentation). FA: formic acid. *Same *m/z* values for non-Ox-TXG oligosaccharides ([M + FA - H]^-^) and C1-Ox-TXG oligosaccharides ([M - H]^-^). Hex = hexaose, Pen = pentaose.

| **9C_non-Ox**  **Peak Nr.** | **Elution time (min)** | ***m/z***  **([M - H]^-^)** | **MS/MS fragments (*m/z*)^a^** | **Annotation** |
| --- | --- | --- | --- | --- |
| 1 | 20.1 - 20.7 | 767.4 | 161 (1), 293 (37), 311 (6), 353 (1), 413 (1), 527 (30), 545 (9), 605 (100),  689 (15), 707 (3), 749 (11) | XXG |
| 2 | 23.4 - 24.5 | 899.3 | 293 (51), 311 (8), 527 (34), 545 (8), 605 (100), 821 (44), 839 (13) | XXX |
| 3 | 28.8 - 30.5 | 1061.4 | 311 (6), 455 (100), 527 (20), 545 (3), 605 (40), 899 (1), 983 (24),  1001 (21), 1043 (5) | XXL |
|  |  |  | 293 (25), 353 (23), 689 (2), 707 (2), 767 (17), 983 (24), 1001 (21),  1043 (5) | XLX |
| 4 | 33.9 - 35.2 | 1223.5 | 293 (4), 311 (2), 353 (25), 455 (100), 689 (14), 707 (8), 767 (60),  1061 (2), 1145 (18), 1163 (58), 1205 (6) | XLL |
| 5 | 36.9 - 37.7 | 1385.7 | 455 (9), 707 (2), 767 (12), 1145 (6), 1163 (8), 1223 (100), 1307 (12),  1325 (50), 1367 (10) | (H_5_P_3_)G |
| 6 | 39.6 - 41.0 | 1385.7 | 293 (2), 353 (9), 455 (46), 473 (2), 851 (10), 869 (12), 929 (100),  1163 (2), 1223 (16), 1307 (20), 1325 (73), 1367 (3) | GXLL |

**Table S2**. List of non-oxidized XG oligosaccharides identified based on fragmentation patterns in CID-MS/MS present in the *Nc*LPMO9C-TXG-digest. Chromatograms, including peak numbers, are shown in **Fig. 5**. Nomenclature (annotation) according to Fry et al. (4).

^a^Relative intensities of MS/MS fragments are shown between brackets

| **9M_non-Ox**  **Peak Nr.** | **Elution time (min)** | ***m/z***  **([M - H]^-^)** | **MS/MS fragments (*m/z*)^a^** | **Annotation** |
| --- | --- | --- | --- | --- |
| 1 | 8.3 - 8.7 | 473.2 | 149 (7), 161 (9), 221 (3), 311 (100), 395 (9), 413 (1), 455 (25) | XG |
|  |  |  | 149 (7), 179 (1), 293 (10), 353 (10), 395 (9), 413 (1), 455 (25) | GX |
| 2 | 12.8 - 13.1 | 605.2 | 149 (6), 293 (100), 311 (14), 353 (1), 527 (20), 545 (7), 587 (5) | XX |
| 3 | 15.3 - 16.2 | 635.3 | 161 (1), 179 (3), 221 (1), 311 (41), 353 (8), 413 (5), 455 (11), 557 (5), 575 (6), 617 (1) | LG |
|  |  |  | 161 (1), 179 (3), 293 (9), 311 (41), 353 (8), 473 (100), 515 (2), 557 (5), 575 (6), 617 (1) | GL |
| 4 | 18.1-19.3 | 767.4 | 149 (1), 161 (1), 293 (30), 311 (7), 413 (1), 455 (1), 527 (26), 545 (9), 605 (100), 689 (13), 707 (3), 749 (3) | XXG |
| 5 | 20.3 - 21.2 | 767.4 | 131 (1), 149 (3), 293 (73), 311 (45), 353 (9), 395 (5), 413 (7), 455 (48), 473 (100), 513 (1), 689 (18), 707 (22), 749 (8) | XGX |
| 6 | 24.2-25.1 | 929.3 | 179 (1), 293 (1), 353 (6), 455 (84), 635 (3), 575 (1), 851 (20), 869 (13), 911 (2) | GLX |
|  |  |  | 161 (1), 221 (2), 353 (6), 455 (84), 689 (6), 707 (8), 767 (100), 851 (20), 869 (13), 911 (2) | XLG |
| 7 | 25.6 - 26.7 | 929.3 | 179 (1), 293 (4), 311 (3), 353 (1), 455 (10), 473 (11), 515 (1), 689 (3), 707 (4), 767 (100), 851 (27), 869 (6), 911 (2) | LXG |
| 8 | 27.5 - 29.0 | 929.3 | 179 (3), 293 (8), 311 (15), 353 (12), 455 (46), 473 (40), 515 (2), 557 (4), 575 (5), 635 (100), 851 (22), 869 (25), 911 (2) | LGX |
| 9 | 30.0 - 31.1 | 1061.4 | 311 (3), 455 (6), 527 (7), 545 (2), 605 (47), 899 (1), 983 (19), 1001 (6), 1043 (5) | XXXG |
|  |  |  | 293 (13), 353 (1), 689 (25), 707 (6), 767 (100), 983 (19), 1001 (6), 1043 (5) | GXXX |
| 10 | 31.2 - 32.2 | 1091.3 | 353 (2), 413 (1), 455 (13), 473 (2), 851 (1), 869 (5), 929 (100), 1013 (12), 1031 (12), 1073 (1) | LLG |
| 11 | 34.9 - 35.7 | 1223.5 | 293 (1), 311 (1), 353 (2), 455 (21), 689 (3), 707 (3), 767 (20), 851 (25), 869 (12), 929 (100), 1061 (15), 1091 (8), 1145 (22), 1163 (17), 1205 (3) | XLGX |
| 12 | 36.6 - 37.2 | 1223.5 | 293 (3), 311 (3), 353 (2), 455 (3), 473 (6), 689 (3), 707 (1), 767 (19), 851 (20), 869 (8), 929 (100), 1061 (1), 1091 (1), 1145 (29), 1163 (30), 1205 (1) | LXGX |
| 13 | 37.5 - 38.2 | 1385.7 | 353 (2), 455 (9), 689 (1), 707 (1), 767 (8), 1145 (5), 1163 (13), 1223 (100), 1307 (20), 1325 (79), 1367 (3) | XLLG |
| 14 | 40.8 - 41.2 | 1385.7 | 353 (1), 455 (4), 473 (1), 851 (2), 869 (1), 929 (26), 1013 (13), 1031 (11), 1091 (100), 1307 (17), 1325 (14), 1367 (1) | LLGX |
| 15 | 41.9 - 42.3 | 1385.7 | 293 (3), 311 (5), 353 (4), 455 (2), 515 (1), 557 (2), 575 (2), 635 (24), 851 (42), 869 (10), 929 (81), 1091 (8), 1223 (3), 1307 (25), 1325 (100), 1367 (1) | LGXL |
| 16 | 43.2 - 44.7 | 1517.6 | n.d. | H_6_P_4_ |
| 17 | > 46.0 | n.d. | n.d. | DP > 10 |

**Table S3**. List of non-oxidized XG oligosaccharides identified based on fragmentation patterns in CID-MS/MS present in the *Nc*LPMO9M-TXG-digest. Chromatograms, including peak numbers, are shown in **Fig. 5**. Nomenclature (annotation) according to Fry et al. (4).

^a^Relative intensities of MS/MS fragments are shown between brackets

**Table S4**. Characterized LPMOs; organism, GenBank accession number, LPMO name (if applicable), Protein Data Bank (PDB) entry (if applicable) and reference (if applicable). Numbers correspond to the numbers shown in the SO phylogenetic tree in **Fig. 9**. # Sequence contains a carbohydrate binding module (CBM).

| **Number** | **Organism** | **GenBank accession number** | **LPMO name** | **PDB entry** | **Reference** |
| --- | --- | --- | --- | --- | --- |
| 1 | *Arthrobotrys oligospora* | EGX44929.1# |  |  |  |
| 2 | *Leptosphaeria maculans* | CBY01974.1 |  |  |  |
| 3 | *Colletotrichum graminicola* | CAQ16208.1 |  |  |  |
| 4 | *Botrytis cinerea* | ATZ55262.1 |  |  |  |
| 5 | *Neurospora crassa* | EAA33178.1 | *Nc*LPMO9M | 4EIS | (5, 6) |
| 6 | *Gloeophyllum trabeum* | AEJ35168.1 |  |  |  |
| 7 | *Gloeophyllum trabeum* | BAV57612.1 | *Gt*LPMO9A-2 |  | (7) |
| 8 | *Thermothelomyces thermophilus (Myceliophthora thermophila)* | ASE05899.1 | *Mt*LPMO9D | 5UFV | (8, 9) |
| 9 | *Thielavia terrestris* | AEO65580.1 |  |  |  |
| 10 | *Heterobasidion irregulare* | ETW87087.1 | *Hi*LPMO9B | 5NNS | (10) |
| 11 | *Heterobasidion parviporum* | AFO72234.1# |  |  |  |
| 12 | *Fusarium venenatum* | CEI62259.1 |  |  |  |
| 13 | *Zymoseptoria tritici* | SMY21959.1 |  |  |  |
| 14 | *Fusarium venenatum* | CEI66075.1 |  |  |  |
| 15 | *Fusarium graminearum* | CEF74460.1 | *Fg*LPMO9A |  | (11) |
| 16 | *Fusarium fujikuroi* | CCT72465.1 |  |  |  |
| 17 | *Podospora anserina* | CAP66744.1 | *Pa*LPMO9D |  | (12) |
| 18 | *Lasiodiplodia theobromae* | CAJ81217.1 |  |  |  |
| 19 | *Neurospora crassa* | EAA29018.1# | NCU07760 |  | (6) |
| 20 | *Podospora anserina* | CAP73254.1# | *Pa*LPMO9A |  | (12) |
| 21 | *Trichoderma reesei (Hypocrea jecorina)* | CAA71999.1# | *Tr*Cel61A (*Hj*LPMO9A) | 5O2W | (13-15) |
| 22 | *Aspergillus oryzae* | BAE60320.1 |  |  |  |
| 23 | *Penicillium rubens Wisconsin* | CAP80988.1# |  |  |  |
| 24 | *Aspergillus niger* | CAK46515.1# |  |  |  |
| 25 | *Botrytis cinerea* | CCD50139.1 |  |  |  |
| 26 | *Fusarium fujikuroi* | CCT72942.1 |  |  |  |
| 27 | *Aspergillus fumigatus* | CAF31975.1 |  |  |  |
| 28 | *Talaromyces piceae* | ATQ35955.1 |  |  |  |
| 29 | *Penicillium oxalicum* | AIO06742.1 |  |  |  |
| 30 | *Aspergillus niger* | CAK38942.1 |  |  |  |

| **Number** | **Organism** | **GenBank accession number** | **LPMO name** | **PDB entry** | **Reference** |
| --- | --- | --- | --- | --- | --- |
| 31 | *Thermoascus aurantiacus* | ACS05720.1 | *Ta*LPMO9A | 2YET | (16) |
| 32 | *Trichoderma reesei (Hypocrea jecorina)* | AAP57753.1 | *Tr*Cel61B | 2VTC | (17) |
| 33 | *Pestalotiopsis sp.* | ANB32143.1 |  |  |  |
| 34 | *Sclerotinia sclerotiorum* | APA12066.1 |  |  |  |
| 35 | *Aspergillus fumigatus* | XP_752040.1 | *Af*AA9_B | 6HAQ | (18) |
| 36 | *Penicillium rubens Wisconsin* | CAP92380.1 |  |  |  |
| 37 | *Aspergillus oryzae* | BAE56764.1 |  |  |  |
| 38 | *Geotrichum candidum* | CDO57961.1 | *Gc*LPMO9B |  | (19) |
| 39 | *Geotrichum candidum* | CDO58049.1 | *Gc*LPMO9A |  | (19) |
| 40 | *Podospora anserina* | CDP30131.1 |  |  |  |
| 41 | *Thermothelomyces thermophilus (Myceliophthora thermophila)* | AEO56016.1 | *Mt*LPMO9J |  | (20) |
| 42 | *Thielavia terrestris* | AEO62422.1 |  |  |  |
| 43 | *Neurospora crassa* | EAA36362.1# | *Nc*LPMO9C | 4D7U | (5, 6, 21) |
| 44 | *Humicola insolens* | CAG27577.1# |  |  |  |
| 45 | *Podospora anserina* | CAP61476.1 | *Pa*LPMO9H |  | (12, 22) |
| 46 | *Serendipita indica* | CCA67659.1# |  |  |  |
| 47 | *Neurospora crassa* | EAA30263.1 | *Nc*LPMO9A | 5FOH | (6, 23, 24) |
| 48 | *Pestalotiopsis sp* | ANB32140.1 | *Ps*LPMO9B |  | (25) |
| 49 | *Sclerotinia sclerotiorum* | APA15869.1# |  |  |  |
| 50 | *Thermothelomyces thermophilus (Myceliophthora thermophila)* | AEO56642.1 | *Mt*LPMO9C |  | (26) |
| 51 | *Neurospora crassa* | CAD21296.1 | *Nc*LPMO9D | 4EIR | (6, 23, 27) |
| 52 | *Pyricularia oryzae* | QBZ61923.1 |  |  |  |
| 53 | *Podospora anserine* | CAP65111.1 |  |  |  |
| 54 | *Collariella virescens* | AST24379.1 | *Cv*AA9A | 5NLT | (28) |
| 55 | *Aspergillus nidulans* | EAA64722.1# |  |  |  |
| 56 | *Lentinus similis* | ALN96977.1 | *Ls*AA9A | 5ACF | (28) |
| 57 | *Rhizophlyctis rosea* | AWM99283.1 |  |  |  |
| 58 | *Rhizophlyctis rosea* | AWM99279.1# |  |  |  |
| 59 | *Scytalidium lignicola* | AYO97682.1# |  |  |  |
| 60 | *Thielavia terrestris* | AEO67395.1# |  |  |  |
| 61 | *Neurospora crassa* | CAE81966.1# | *Nc*LPMO9J |  | (2) |
| 62 | *Podospora anserina* | CAP68375.1# | *Pa*LPMO9B |  | (12) |

| **Number** | **Organism** | **GenBank accession number** | **LPMO name** | **PDB entry** | **Reference** |
| --- | --- | --- | --- | --- | --- |
| 63 | *Thermothelomyces thermophilus (Myceliophthora thermophila)* | AON76800.1# | *Mt*LPMO9B |  | (26) |
| 64 | *Neurospora crassa* | EAA26873.2# | *Nc*LPMO9E |  | (6) |
| 65 | *Botrytis cinerea* | ATZ48364.1 |  |  |  |
| 66 | *Thermothelomyces thermophilus (Myceliophthora thermophila)* | AEO55652.1 | *Mt*LPMO9I |  | (3) |
| 67 | *Podospora anserina* | CAP61048.1 |  |  |  |
| 68 | *Zymoseptoria tritici* | SMR44834.1 |  |  |  |
| 69 | *Podospora anserina* | CAP67466.1 |  |  |  |
| 70 | *Podospora comata* | VBB71335.1# |  |  |  |
| 71 | *Podospora comata* | VBB79368.1# |  |  |  |
| 72 | *Pyricularia oryzae* | XP 003717521.1 |  |  |  |
| 73 | *Aspergillus nidulans* | EAA63617.1 | AN3046 |  | (29) |
| 74 | *Fusarium graminearum* | CEF78876.1 |  |  |  |
| 75 | *Phanerochaete chrysosporium* | BAL43430.1 | *Pc*LPMO9D | 4B5Q | (30) |
| 76 | *Thielavia terrestris* | XP_003657366.1 | *Tt*LPMO9E | 3EII | (31) |
| 77 | *Thermothelomyces thermophilus (Myceliophthora thermophila)* | AEO61304.1 |  |  |  |
| 78 | *Neurospora crassa* | CAD70347.1 | *Nc*LPMO9F | 4QI8 | (5) |
| 79 | *Coprinopsis cinerea* | CAG27578.1 |  |  |  |
| 80 | *Malbranchea cinnamomea* | QCQ84225.1 |  |  |  |
| 81 | *Thermothelomyces thermophilus* | AEO60271.1 | MYCTH_112089 |  | (6) |
| 82 | *Pestalotiopsis sp* | ANB32141.1 | *Ps*LPMO9A |  | (25) |
| 83 | *Neurospora crassa* | EAA34466.1# | NCU00836 |  | (6) |
| 84 | *Podospora anserina* | CAP67740.1# | *Pa*LPMO9E |  | (12) |
| 85 | *Podospora comata* | VBB79669.1 |  |  |  |

**References**

1. Domon B, Costello CE. A systematic nomenclature for carbohydrate fragmentations in FAB-MS/MS spectra of glycoconjugates. Glycoconjugate J. 1988;5:397-409.

2. Kittl R, Kracher D, Burgstaller D, Haltrich D, Ludwig R. Production of four *Neurospora crassa* lytic polysaccharide monooxygenases in *Pichia pastoris* monitored by a fluorimetric assay. Biotechnol Biofuels. 2012;5.

3. Sun P, Frommhagen M, Kleine Haar M, van Erven G, Bakx E, van Berkel W, et al. Mass spectrometric fragmentation patterns discriminate C1-and C4-oxidised cello-oligosaccharides from their non-oxidised and reduced forms. Carbohydr Polym. 2020;234:115917.

4. Fry SC, York WS, Albersheim P, Darvill A, Hayashi T, Joseleau JP, et al. An unambiguous nomenclature for xyloglucan-derived oligosaccharides. Physiol Plant. 1993;89:1-3.

5. Li X, Beeson IV WT, Phillips CM, Marletta MA, Cate JH. Structural basis for substrate targeting and catalysis by fungal polysaccharide monooxygenases. Structure. 2012;20:1051-61.

6. Vu VV, Beeson WT, Phillips CM, Cate JH, Marletta MA. Determinants of regioselective hydroxylation in the fungal polysaccharide monooxygenases. J Am Chem Soc. 2014;136:562-5.

7. Kojima Y, Varnai A, Ishida T, Sunagawa N, Petrovic DM, Igarashi K, et al. A lytic polysaccharide monooxygenase with broad xyloglucan specificity from the brown-rot fungus *Gloeophyllum trabeum* and its action on cellulose-xyloglucan complexes. Appl Environ Microbiol. 2016;82:6557-72.

8. Span EA, Suess DLM, Deller MC, Britt RD, Marletta MA. The role of the secondary coordination sphere in a fungal polysaccharide monooxygenase. ACS Chem. Biol. 2017;12:1095-103.

9. Frommhagen M, Westphal AH, Hilgers R, Koetsier MJ, Hinz SWA, Visser J, et al. Quantification of the catalytic performance of C1-cellulose-specific lytic polysaccharide monooxygenases. Appl Microbiol Biotechnol. 2017;102:1281-95.

10. Liu B, Kognole AA, Wu M, Westereng B, Crowley MF, Kim S, et al. Structural and molecular dynamics studies of a C1-oxidizing lytic polysaccharide monooxygenase from *Heterobasidion irregulare* reveal amino acids important for substrate recognition. FEBS J. 2018;285:2225-42.

11. Nekiunaite L, Petrovic DM, Westereng B, Vaaje-Kolstad G, Hachem MA, Varnai A, et al. *Fg*LPMO9A from *Fusarium graminearum* cleaves xyloglucan independently of the backbone substitution pattern. FEBS Lett. 2016;590:3346-56.

12. Bennati-Granier C, Garajova S, Champion C, Grisel S, Haon M, Zhou S, et al. Substrate specificity and regioselectivity of fungal AA9 lytic polysaccharide monooxygenases secreted by *Podospora anserina*. Biotechnol Biofuels. 2015;8:90.

13. Pierce BC, Agger JW, Zhang ZH, Wichmann J, Meyer AS. A comparative study on the activity of fungal lytic polysaccharide monooxygenases for the depolymerization of cellulose in soybean spent flakes. Carbohydr Res. 2017;449:85-94.

14. Hansson H, Karkehabadi S, Mikkelsen N, Douglas NR, Kim S, Lam A, et al. High-resolution structure of a lytic polysaccharide monooxygenase from *Hypocrea jecorina* reveals a predicted linker as an integral part of the catalytic domain. J Biol Chem. 2017;292:19099-109.

15. Gusakov AV, Bulakhov AG, Demin IN, Sinitsyn AP. Monitoring of reactions catalyzed by lytic polysaccharide monooxygenases using highly-sensitive fluorimetric assay of the oxygen consumption rate. Carbohydr Res. 2017;452:156-61.

16. Petrovic DM, Bissaro B, Chylenski P, Skaugen M, Sorlie M, Jensen MS, et al. Methylation of the N-terminal histidine protects a lytic polysaccharide monooxygenase from auto-oxidative inactivation. Protein Sci. 2018;27:1636-50.

17. Karkehabadi S, Hansson H, Kim S, Piens K, Mitchinson C, Sandgren M. The first structure of a glycoside hydrolase family 61 member, Cel61B from *Hypocrea jecorina*, at 1.6 angstrom resolution. J Mol Biol. 2008;383:144-54.

18. Lo Leggio L, Weihe CD, Poulsen JCN, Sweeney M, Rasmussen F, Lin J, et al. Structure of a lytic polysaccharide monooxygenase from *Aspergillus fumigatus* and an engineered thermostable variant. Carbohydr Res. 2018;469:55-9.

19. Ladeveze S, Haon M, Villares A, Cathala B, Grisel S, Herpoel-Gimbert I, et al. The yeast *Geotrichum candidum* encodes functional lytic polysaccharide monooxygenases. Biotechnol Biofuels. 2017;10:215.

20. Kadowaki MA, Várnai A, Jameson J-K, Leite AE, Costa-Filho AJ, Kumagai PS, et al. Functional characterization of a lytic polysaccharide monooxygenase from the thermophilic fungus *Myceliophthora thermophila*. PLOS ONE. 2018;13:e0202148.

21. Agger JW, Isaksen T, Varnai A, Vidal-Melgosa S, Willats WG, Ludwig R, et al. Discovery of LPMO activity on hemicelluloses shows the importance of oxidative processes in plant cell wall degradation. Proc Natl Acad Sci USA. 2014;111:6287-92.

22. Fanuel M, Garajova S, Ropartz D, McGregor N, Brumer H, Rogniaux H, et al. The *Podospora anserina* lytic polysaccharide monooxygenase *Pa*LPMO9H catalyzes oxidative cleavage of diverse plant cell wall matrix glycans. Biotechnol Biofuels. 2017;10:63.

23. Petrovic DM, Varnai A, Dimarogona M, Mathiesen G, Sandgren M, Westereng B, et al. Comparison of three seemingly similar lytic polysaccharide monooxygenases from *Neurospora crassa* suggests different roles in plant biomass degradation. J Biol Chem. 2019;294:15068-81.

24. Westereng B, Arntzen MO, Aachmann FL, Varnai A, Eijsink VG, Agger JW. Simultaneous analysis of C1 and C4 oxidized oligosaccharides, the products of lytic polysaccharide monooxygenases acting on cellulose. J Chromatogr A. 2016;1445:46-54.

25. Patel I, Kracher D, Ma S, Garajova S, Haon M, Faulds CB, et al. Salt-responsive lytic polysaccharide monooxygenases from the mangrove fungus *Pestalotiopsis sp* NCi6. Biotechnol Biofuels. 2016;9.

26. Frommhagen M, Koetsier MJ, Westphal AH, Visser J, Hinz SW, Vincken J-P, et al. Lytic polysaccharide monooxygenases from *Myceliophthora thermophila* C1 differ in substrate preference and reducing agent specificity. Biotechnol biofuels. 2016;9:186.

27. Bodenheimer AM, O'Dell WB, Stanley CB, Meilleur F. Structural studies of *Neurospora crassa* LPMO9D and redox partner CDHIIA using neutron crystallography and small-angle scattering. Carbohydr Res. 2017;448:200-4.

28. Simmons TJ, Frandsen KEH, Ciano L, Tryfona T, Lenfant N, Poulsen JC, et al. Structural and electronic determinants of lytic polysaccharide monooxygenase reactivity on polysaccharide substrates. Nat Commun. 2017;8:1064.

29. Jagadeeswaran G, Gainey L, Prade R, Mort AJ. A family of AA9 lytic polysaccharide monooxygenases in *Aspergillus nidulans* is differentially regulated by multiple substrates and at least one is active on cellulose and xyloglucan. Appl Microbiol Biotechnol. 2016;100:4535-47.

30. Wu M, Beckham GT, Larsson AM, Ishida T, Kim S, Payne CM, et al. Crystal structure and computational characterization of the lytic polysaccharide monooxygenase GH61D from the Basidiomycota fungus *Phanerochaete chrysosporium*. J Biol Chem. 2013;288:12828-39.

31. Cannella D, Mollers KB, Frigaard NU, Jensen PE, Bjerrum MJ, Johansen KS, et al. Light-driven oxidation of polysaccharides by photosynthetic pigments and a metalloenzyme. Nat Commun. 2016;7:11134.
