# Supplementary material for: Configuration of active site segments in lytic polysaccharide monooxygenases steers oxidative xyloglucan degradation
Source: Biotechnol Biofuels. 2020 May 29;13:95. doi: 10.1186/s13068-020-01731-x (PMC7257166; doi:10.1186/s13068-020-01731-x)
Supplement: Supplementary file 3 — Additional file 3. Structure-based amino acid “segments only” sequence alignment of AA9 LPMOs. [file 13068_2020_1731_MOESM3_ESM.pdf]

Number	Organism	GenBank asscession number	LPMO name
1	*Arthrobotrys oligospora*	EGX44929.1#	
2	*Leptosphaeria maculans*	CBY01974.1	
3	*Colletotrichum graminicola*	CAQ16208.1	
4	*Botrytis cinerea*	ATZ55262.1	
5	*Neurospora crassa*	EAA33178.1	*Nc* LPMO9M
6	*Gloeophyllum trabeum*	AEJ35168.1	
7	*Gloeophyllum trabeum*	BAV57612.1	*Gt* LPMO9A-2
8	*Thermothelomyces thermophilus*	ASE05899.1	*Mt* LPMO9D
9	*Thielavia terrestris*	AEO65580.1	
10	*Heterobasidion irregulare*	ETW87087.1	*Hi* LPMO9B
11	*Heterobasidion parviporum*	AFO72234.1#	
12	*Fusarium venenatum*	CEI62259.1	
13	*Zymoseptoria tritici*	SMY21959.1	
14	*Fusarium venenatum*	CEI66075.1	
15	*Fusarium graminearum*	CEF74460.1	*Fg* LPMO9A
16	*Fusarium fujikuroi*	CCT72465.1	
17	*Podospora anserina*	CAP66744.1	*Pa* LPMO9D
18	*Lasiodiplodia theobromae*	CAJ81217.1	
19	*Neurospora crassa*	EAA29018.1#	NCU07760
20	*Podospora anserina*	CAP73254.1#	*Pa* LPMO9A
21	*Trichoderma reesei*	CAA71999.1#	*Tr* Cel61A
22	*Aspergillus oryzae*	BAE60320.1	
23	*Penicillium rubens Wisconsin*	CAP80988.1#	
24	*Aspergillus niger*	CAK46515.1#	
25	*Botrytis cinerea*	CCD50139.1	
26	*Fusarium fujikuroi*	CCT72942.1	
27	*Aspergillus fumigatus*	CAF31975.1	
28	*Talaromyces piceae*	ATQ35955.1	
29	*Penicillium oxalicum*	AIO06742.1	
30	*Aspergillus niger*	CAK38942.1	
31	*Thermoascus aurantiacus*	ACS05720.1	*Ta* LPMO9A
32	*Trichoderma reesei*	AAP57753.1	*Tr* Cel61B
33	*Pestalotiopsis sp.*	ANB32143.1	
34	*Sclerotinia sclerotiorum*	APA12066.1	
35	*Aspergillus fumigatus*	XP 752040.1	*Af* AA9 B
36	*Penicillium rubens Wisconsin*	CAP92380.1	
37	*Aspergillus oryzae*	BAE56764.1	
38	*Geotrichum candidum*	CDO57961.1	*Gc* LPMO9B
39	*Geotrichum candidum*	CDO58049.1	*Gc* LPMO9A
40	*Podospora anserina*	CDP30131.1	
41	*Thermothelomyces thermophilus*	AEO56016.1	*Mt* LPMO9J
42	*Thielavia terrestris*	AEO62422.1	
43	*Neurospora crassa*	EAA36362.1#	*Nc* LPMO9C
44	*Humicola insolens*	CAG27577.1#	
45	*Podospora anserina*	CAP61476.1	*Pa* LPMO9H
46	*Serendipita indica*	CCA67659.1#	
47	*Neurospora crassa*	EAA30263.1	*Nc* LPMO9A
48	*Pestalotiopsis sp*	ANB32140.1	*Ps* LPMO9B
49	*Sclerotinia sclerotiorum*	APA15869.1#	
50	*Thermothelomyces thermophilus*	AEO56642.1	*Mt* LPMO9C
51	*Neurospora crassa*	CAD21296.1	*Nc* LPMO9D
52	*Pyricularia oryzae*	QBZ61923.1	
53	*Podospora anserine*	CAP65111.1	
54	*Collariella virescens*	AST24379.1	*Cv* AA9A
55	*Aspergillus nidulans*	EAA64722.1#	
56	*Lentinus similis*	ALN96977.1	*Ls* AA9A
57	*Rhizophlyctis rosea*	AWM99283.1	
58	*Rhizophlyctis rosea*	AWM99279.1#	
59	*Scytalidium lignicola*	AYO97682.1#	
60	*Thielavia terrestris*	AEO67395.1#	
61	*Neurospora crassa*	CAE81966.1#	*Nc* LPMO9J
62	*Podospora anserina*	CAP68375.1#	*Pa* LPMO9B
63	*Thermothelomyces thermophilus*	AON76800.1#	*Mt* LPMO9B
64	*Neurospora crassa*	EAA26873.2#	*Nc* LPMO9E
65	*Botrytis cinerea*	ATZ48364.1	
66	*Thermothelomyces thermophilus*	AEO55652.1	*Mt* LPMO9I
67	*Podospora anserina*	CAP61048.1	
68	*Zymoseptoria tritici*	SMR44834.1	
69	*Podospora anserina*	CAP67466.1	
70	*Podospora comata*	VBB71335.1#	
71	*Podospora comata*	VBB79368.1#	
72	*Pyricularia oryzae*	XP 003717521.1	
73	*Aspergillus nidulans*	EAA63617.1	AN3046
74	*Fusarium graminearum*	CEF78876.1	
75	*Phanerochaete chrysosporium*	BAL43430.1	*Pc* LPMO9D
76	*Thielavia terrestris*	XP 003657366.1	*Tt* LPMO9E
77	*Thermothelomyces thermophilus*	AEO61304.1	
78	*Neurospora crassa*	CAD70347.1	*Nc* LPMO9F
79	*Coprinopsis cinerea*	CAG27578.1	
80	*Malbranchea cinnamomea*	QCQ84225.1	
81	*Thermothelomyces thermophilus*	AEO60271.1	MYCTH_112089
82	*Pestalotiopsis sp*	ANB32141.1	*Ps* LPMO9A
83	*Neurospora crassa*	EAA34466.1#	NCU00836
84	*Podospora anserina*	CAP67740.1#	*Pa* LPMO9E
85	*Podospora comata*	VBB79669.1	

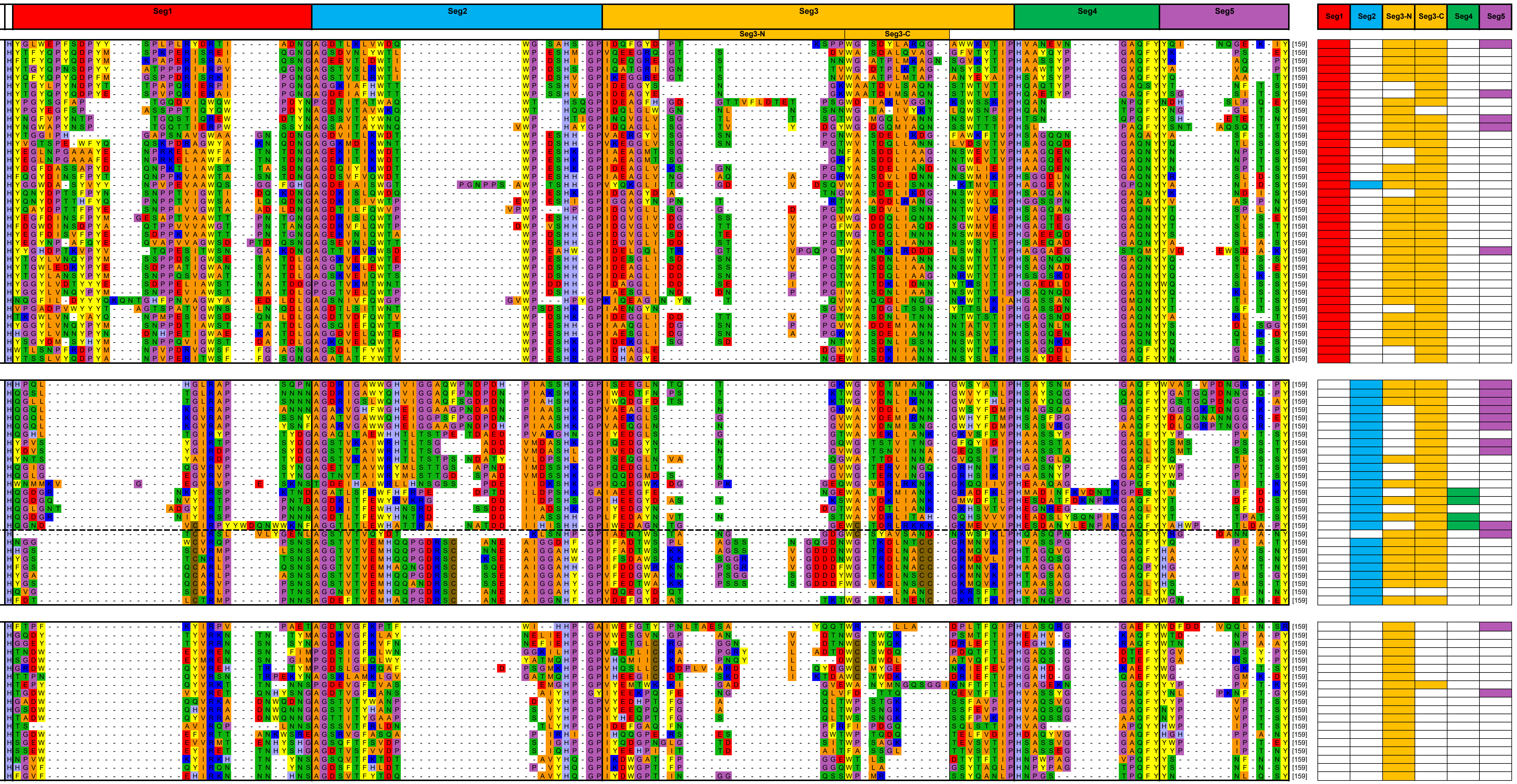

Sequence contains a Carbohydrate Binding Module (CBM)

Solid line indicates the main clusters (+Seg1-Seg2, -Seg1+Seg2 and -Seg1-Seg2) and dashed line indicates one sub-cluster (-Seg1+Seg2+Seg3)

Color codes	
**Amino acids**	
H	lavender
K,R	blue
D,E	red
S,T,N,Q	green
A,V,L,I,M	orange
F,W,Y	yellow
C	brown
P,G	purple
**Segments**	
Seg1	red
Seg2	light blue
Seg3	orange
Seg4	green
Seg5	purple
