# Supplementary material for: Configuration of active site segments in lytic polysaccharide monooxygenases steers oxidative xyloglucan degradation
Source: Biotechnol Biofuels. 2020 May 29;13:95. doi: 10.1186/s13068-020-01731-x (PMC7257166; doi:10.1186/s13068-020-01731-x)
Supplement: Supplementary file 2 — Additional file 2. Structure-based amino acid “full sequence” alignment of AA9 LPMOs. [file 13068_2020_1731_MOESM2_ESM.pdf]

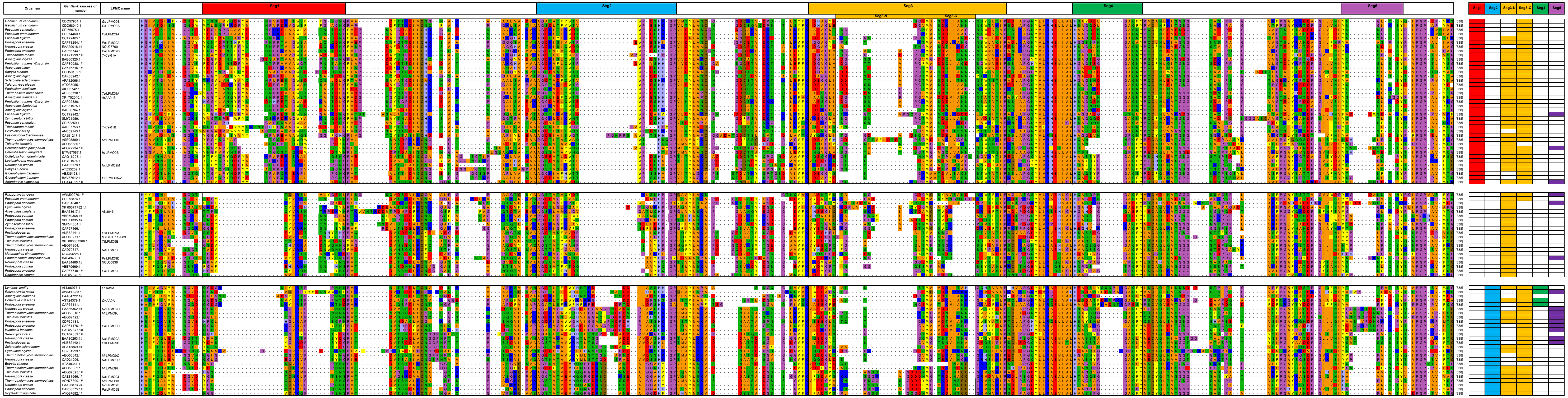

Sequence contains a Carbohydrate Binding Module (CBM)

Solid line indicates the main clusters (+Seq1-Seg2, -Seg1+Seg2 and -Seg1-Seg2) and dashed line indicates one sub-cluster (-Seg1+Seg2+Seg3)

Color codes	
**Amino acids**	
H	
K,R	
D,E	
S,T,N,Q	
A,V,L,I,M	
F,W,Y	
C	
P,G	
**Segments**	
Seg1	
Seg2	
Seg3	
Seg4	
Seg5	
